# Anti-Neuroinflammatory Components from *Clausena lenis* Drake

**DOI:** 10.3390/molecules27061971

**Published:** 2022-03-18

**Authors:** Si-Si Zhu, Yi-Fan Zhang, Meng Ding, Ke-Wu Zeng, Peng-Fei Tu, Yong Jiang

**Affiliations:** State Key Laboratory of Natural and Biomimetic Drugs, School of Pharmaceutical Sciences, Peking University, No. 38 Xueyuan Road, Haidian District, Beijing 100191, China; zsszmh@163.com (S.-S.Z.); yifan9986@163.com (Y.-F.Z.); stillmedingmeng@163.com (M.D.); zkw@bjmu.edu.cn (K.-W.Z.); pengfeitu@bjmu.edu.cn (P.-F.T.)

**Keywords:** *Clausena lenis* Drake, alkaloid, coumarin, anti-neuroinflammation, BV-2 cells

## Abstract

*Clausena lenis* Drake (*C. lenis*) is a folk medicinal herb to treat influenza, colds, bronchitis, and malaria. The 95% and 50% ethanol extract of *C. lenis* showed significant nitric oxide (NO) inhibition activity in BV-2 microglial cells stimulated by lipopolysaccharide (LPS). Bio-guided isolation of the active extract afforded five new compounds, including a chlorine-containing furoquinoline racemate, (±)-claulenine A (**1**), an amide alkaloid, claulenine B (**2**), a prenylated coumarin, claulenin A (**3**), a furocoumarin glucoside, clauleside A (**4**), and a multi-prenylated *p*-hydroxybenzaldehyde, claulenin B (**5**), along with 33 known ones. Their structures were determined via spectroscopic methods, and the absolute configurations of new compounds were assigned via the electronic circular dichroism (ECD) calculations and single-crystal X-ray diffraction analysis. Compounds **2**, **23**, **27**, **28**, **33**, and **34** showed potent anti-neuroinflammatory effects on LPS-induced NO production in BV-2 microglial cells, with IC_50_ values in the range of 17.6–40.9 μM. The possible mechanism was deduced to interact with iNOS through molecular docking.

## 1. Introduction

Neuroinflammation generally refers to an inflammatory response in the brain or spinal cord and has a pivotal role in the pathogenesis of neurodegenerative diseases, such as Alzheimer’s disease (AD) [1] and Parkinson’s disease (PD) [2,3]. This inflammation is mediated by cytokines, chemokines, reactive oxygen species, and secondary messengers produced by resident central glial cells (microglia and astrocytes), endothelial cells, and immune cells of peripheral origin [4]. Among them, microglia, as innate immune cells of the central nervous system, are major participants in neuroinflammation [1] and have various neuroimmunological functions in the central nervous system under normal and pathological conditions [5].

Phytochemicals, as the main components of natural products, have a variety of pharmacological effects, including anti-inflammation, neuroprotection, anti-cancer, and metabolism regulation [6,7,8,9]. In recent years, some compounds derived from *Clausena* species have been revealed to present potential anti-neuroinflammatory activities by inhibiting the nitric oxide (NO) production in lipopolysaccharide (LPS)-induced BV-2 microglial cells [10,11,12].

**Figure 1:**
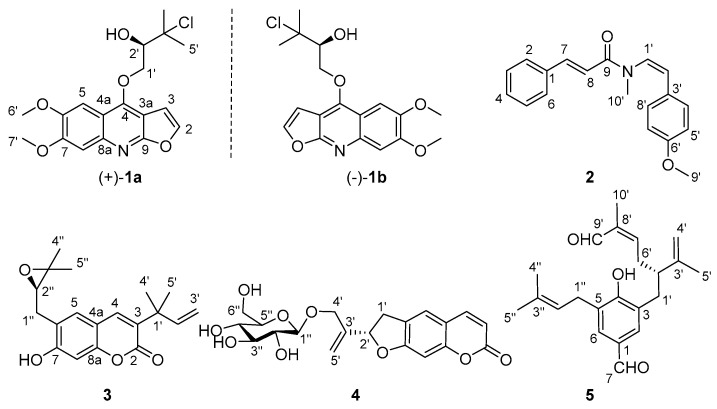
Chemical structures of compounds **1**−**5**.

*Clausena lenis* Drake (*C. lenis*) belongs to the genus *Clausena* of Rutaceae family, distributed mainly in the Hainan, southern Guangxi, and Yunnan provinces of China [13]. It has been used as a folk medicine for the treatment of influenza, colds, bronchitis, and malaria. Alkaloids and coumarins are preliminarily verified to be its main constituents [14,15,16,17,18,19]. During our search for anti-neuroinflammatory components from *Clausena* and its closely related *Murraya* species [20,21,22], the 95% and 50% ethanol extract of *C. lenis* showed significant NO inhibition activity in BV-2 microglial cells stimulated by LPS with 73.5% inhibition rate at 80 ug/mL. To trace the potential anti-neuroinflammatory compounds, the ethanol extract of *C. lenis* was chemically investigated to afford 38 compounds, including five new compounds, namely (±)-claulenine A (**1**), claulenine B (**2**), claulenin A (**3**), clauleside A (**4**), and claulenin B (**5**) (Figure 1). (±)-Claulenine A (**1**) is a racemate of furoquinoline containing chlorine, which is rare in phytochemicals. Herein, the isolation and structure elucidation of the new compounds and the inhibitory effects on LPS-induced NO of the isolates were described. Moreover, the interactions between the bioactive compounds and iNOS via molecular docking were also reported.

## 2. Results

### 2.1. Structural Elucidation

**Figure 2:**
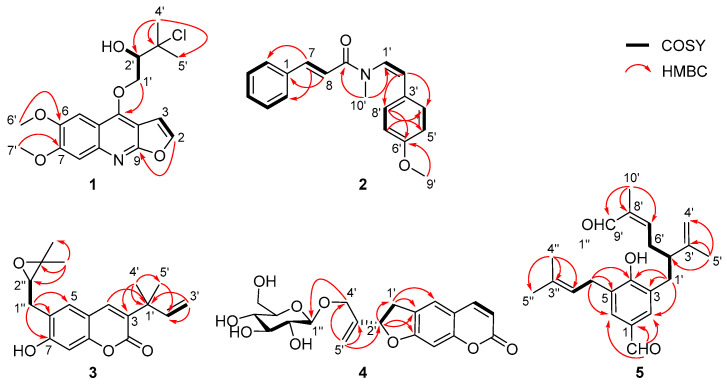
Key HMBC correlations of compounds **1**−**5**.

(±)-Claulenine A (**1**) was obtained as a white solid, ${\left[\alpha \right]}_{D}^{25}$ 0 (*c* 0.12, MeOH). The HR-ESI-MS data indicated the presence of one chlorine in the molecule from the relative abundance (∼1/3) of the isotope peaks observed at *m*/*z* 366.1101 and 368.1077, corresponding to a molecular formula of C_18_H_20_NO_5_Cl (calcd for C_18_H_21_NO_5_Cl, 366.1108; mass error −1.9 ppm), with nine degrees of unsaturation. Analysis of the ^1^H-NMR data (Table 1) revealed the presence of a trisubstituted furoquinoline, which was indicated by the typical adjacent protons of a furan moiety at *δ*_H_ 7.59 (1H, d, *J* = 2.7 Hz, H-2) and 7.00 (1H, d, *J* = 2.7 Hz, H-3) [23] and two aromatic singlet signals at *δ*_H_ 7.45 and 7.34. Meanwhile, two methoxys (*δ*_H_ 4.03 and 4.00), two methyls (both *δ*_H_ 1.77), an oxymethylene [*δ*_H_ 4.93 (1H, dd, *J* = 9.8, 3.7 Hz) and 4.75 (1H, dd, *J* = 9.8, 6.8 Hz)], and an oxymethine [*δ*_H_ 4.18 (1H, dd, *J* = 6.8, 3.7 Hz)] signals were also observed in the ^1^H NMR data (Table 1). In the ^13^C-NMR data of **1**, there were 18 carbon resonances, comprising two methyls, two methoxyls, one methylene, five methines (four olefinic and one oxygenated aliphatic), and eight quaternary carbons, which were very similar to those of (2*S*)-1-[(6,7-dimethoxyfuro[2,3-*b*]quinolin-4-yl)oxy]-3-methyl-butane-2,3-diol [24]. Considering the molecular formula of **1**, one of the two hydroxy groups of (2*S*)-1-[(6,7-dimethoxyfuro[2,3-*b*]quinolin-4-yl)oxy]-3-methyl-butane-2,3-diol was deduced to be substituted by a chlorine atom in **1**. The above deductions were further confirmed by 2D NMR experiments (Figure 2). The two methoxy groups showed HMBC cross-peaks with the carbon at *δ*_C_ 148.1 and 152.8, respectively, locating them at positions C-6 and C-7; H_2_-1′ [*δ*_H_ 4.93 (dd, *J* = 9.8, 3.7 Hz) and 4.75 (dd, *J* = 9.8, 6.8 Hz)] presented an HMBC correlation to C-4 (*δ*_C_ 154.4), suggesting that the oxygenated prenyl unit was linked to C-4 of furoquinoline via an oxygen bridge. In addition, the chlorine at C-3′ and the hydroxy group at C-2′ were indicated by the key COSY correlations (Figure S11, Supporting Information) of –OH [*δ*_H_ 8.12 (1H, d, *J* = 6.2 Hz)], H-2′ [*δ*_H_ 4.58 (1H, ddd, *J* = 7.4, 6.2, 2.8 Hz)], and H-1′ [*δ*_H_ 5.35 (1H, dd, *J* = 9.6, 2.8 Hz), 5.11 (1H, dd, *J* = 9.6, 7.4 Hz)] in C_5_D_5_N. Thus, the planar structure of **1** was established as shown (Figure 1) and denominated as claulenine A.

**Figure 3:**
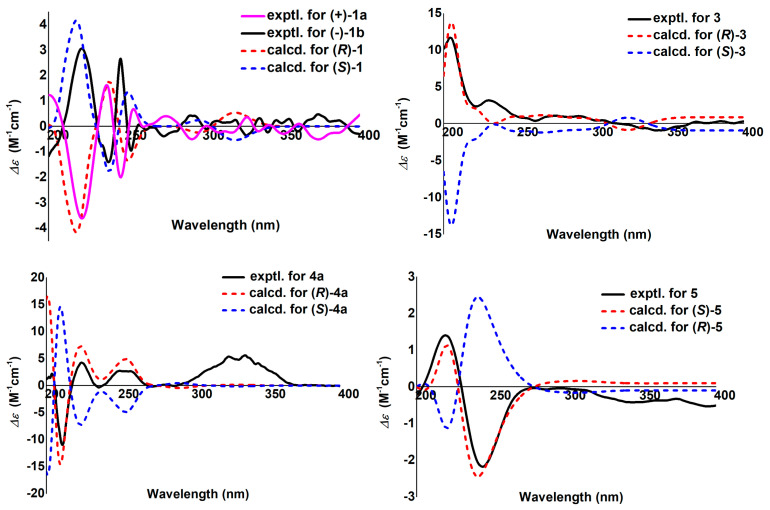
Experimental and calculated ECD spectra of **1**, **3**, **4a**, and **5**.

**Figure 4:**
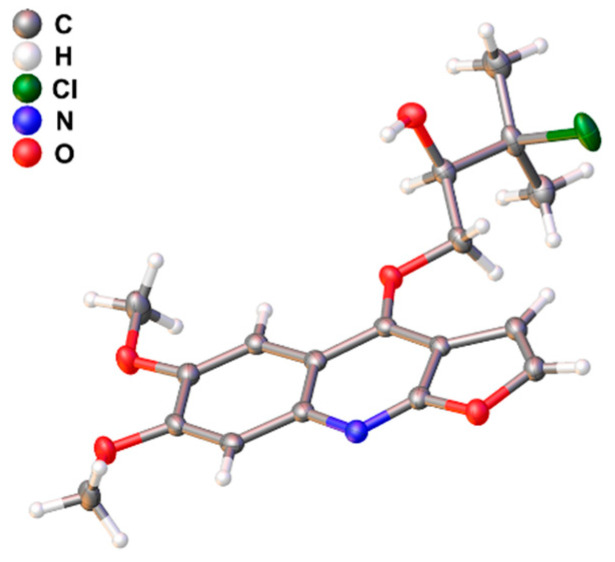
Plot of X-ray crystallographic data for (+)-**1a**.

However, **1** was isolated as a raceme indicated by its zero specific rotation value and no Cotton effects in the electronic circular dichroism (ECD) spectrum. Further HPLC separation on chiral phase afforded the enantiomers of (+)-**1a** and (–)-**1b** (Figure S14, Supporting Information). To clarify their absolute configurations, ECD calculations were utilized, and the results showed that the ECD spectrum of (*R*)-**1** matched well with the experimental curve of (+)-**1a** (Figure 3). This conclusion was finally confirmed by the single-crystal X-ray diffraction for (+)-**1a** using Cu Kα radiation (Figure 4). Considering that there are fewer phytochemicals containing chlorine atoms, a freshly prepared methanol extract of the title plant was detected by using the multiple reaction monitoring (MRM) mode of UPLC/Qtrap-MS/MS, and the results suggested that **1** comes from natural source (Figure S15, Supporting Information).

Claulenine B (**2**) was obtained as a yellow oil, and its molecular formula was determined as C_19_H_19_NO_2_ from the HR-ESI-MS ion at m/z 294.1490 [M + H]^+^ (calcd for C_19_H_20_NO_2_, 294.1494; mass error −1.4 ppm) and ^13^C-NMR data. In the ^1^H NMR spectrum, except for the up-field shifts at *δ*_H_ 3.78 (–OCH_3_) and 3.10 (–NCH_3_), the remaining signals were in the aromatic region (*δ*_H_ 6.2–7.7). The ^13^C NMR data displayed 19 carbons including a signal of amide (*δ*_C_ 166.7). Careful analysis of ^1^H and ^13^C NMR data revealed that the signals of **2** resembled those of anhydromarmeline [25], except that the prenyl moiety was replaced by a methoxy group and an additional methyl positioned at the nitrogen atom in **2**. The HMBC correlations (Figure 2) of –OCH_3_/C-6′ (*δ*_C_ 159.5) and –NCH_3_/C-1′ (*δ*_C_ 127.1), C-9 (*δ*_C_ 166.7) supported the deduction. Besides, the coupling constant of olefinic protons (*J* = 15.5 Hz for H-7 and H-8; *J* = 8.6 Hz for H-1′ and H-2′) pointed out that they were *E*- and *Z*-oriented, respectively. Hence, the gross structure of claulenine B (**2**) was depicted as given (Figure 1).

Claulenin A (**3**), a yellow oil, gave a molecular formula of C_19_H_22_O_4_ (nine degrees of unsaturation), as established by the [M + H]^+^ ion at *m*/*z* 315.1594 (calcd for C_19_H_23_O_4_, 315.1596; mass error −0.6 ppm) in the HR-ESI-MS. The UV spectrum appeared maximum absorptions at 335, 298, 250, and 227 nm, which is typical of the coumarin nucleus [26]. The ^1^H NMR data of **3** (Table 1) exhibited four methyl singlets (*δ*_H_ 1.22, 1.35, 1.46, and 1.46) and three aromatic/olefinic protons [*δ*_H_ 7.47 (s), 7.19 (s), and 6.70 (s)]. The ^13^C-NMR and HSQC spectra displayed 19 carbons classified into four methyls, two methylenes (one aliphatic and one olefinic), five methines (one oxygenated aliphatic and four olefinic), and eight quaternary (one ester carbonyl, two aliphatic, and five olefinic, including two oxygenated) carbons. The ^1^H and ^13^C-NMR data of **3** bore a close resemblance to those of gravelliferone A [19], except that the methoxyl at C-7 in gravelliferone A was replaced by a hydroxy in **3**, which was supported by the molecular formula of **3** and the chemical shift of C-7 (*δ*_C_ 162.4). Therefore, the planar structure of **3** was deduced.

To clarify the absolute configuration of the only chiral center in **3**, ECD calculation was performed. The ECD experimental curve was in good agreement with the calculated one of (*R*)-**3** (Figure 3). Taken together, the structure of **3** was finally assigned and named claulenin A.

Clauleside A (**4**) exhibited an [M − H]^−^ ion at m/z 405.1187 in the HR-ESI-MS, corresponding to a molecular formula of C_20_H_22_O_9_ (calcd for C_20_H_21_O_9_, 405.1186; mass error 0.2 ppm). The UV spectrum is similar to that of **3**, indicating that **4** is also a coumarin derivative. Inspection of ^1^H-NMR and ^13^C-NMR data (Table 1) found a set of characteristic glucosyl signals [*δ*_H_ 4.23 (1H, d, *J* = 7.8 Hz), 3.81 (1H, d, *J* = 11.9 Hz), 3.60 (1H, dd, *J* = 11.9, 4.9 Hz), 3.16–3.38 (4H, m); *δ*_C_ 103.6, 78.0, 78.0, 75.0, 71.6, 62.7], which identified **4** as a coumarin glucoside. Further analysis of NMR data of the aglycone moiety showed that they were basically consistent with those of scataccanol [27]. The HMBC correlations (Figure 2) of H_2_-4′ [*δ*_H_ 4.44 (1H, d, *J* = 12.8 Hz), 4.19 (1H, d, *J* = 12.8 Hz)] to C-1″ (*δ*_C_ 103.6) deduced the linkage of the glucosyl moiety at the C-4′.

The *β*-d-glucose was demonstrated via analysis of the coupling constant (7.8 Hz) of the anomeric proton [28] and the aryl thiocarbamate derivate of the hydrolyte by HPLC (Figure S40, Supporting Information). The absolute configuration of aglycone moiety (**4a**) was assigned as (2′*R*) by comparison of the experimental and calculated ECD curves (Figure 3). Finally, **4** was established as scataccanol 4′-*O*-*β*-d-glucopyranoside and referred to as clauleside A.

Claulenin B (**5**) was isolated as a yellow oil. Its positive-ion HR-ESI-MS data at *m*/*z* 363.1933 [M + Na]^+^ (calcd for C_22_H_28_O_3_Na, 363.1936; mass error −0.8 ppm) established a molecular formula of C_22_H_28_O_3_, with nine indices of hydrogen deficiency. The ^1^H NMR data (Table 1) of **5** showed signals of two aromatic protons [*δ*_H_ 7.52 (2H, br s)] and four methyls [*δ*_H_ 1.70, 1.71, 1.82, and 1.84 (each 3H, s)]. Moreover, the ^13^C-NMR (Table 1) and HSQC (Figure S47, Supporting Information) spectra revealed 22 carbon signals, including two aldehyde-carbonyls (*δ*_C_ 195.4 and 191.4), seven aromatic/olefinic quaternary carbons, five methines (one aliphatic and four olefinic), four methylenes (three aliphatic and one olefinic), and four methyls. The presence of three prenyl derivative units in **5** was illustrated through the HMBC correlations (Figure 2) of H_3_-5′ to C-2′ (*δ*_C_ 46.4), C-3′ (*δ*_C_ 146.2), C-4′ (*δ*_C_ 112.8), H_3_-10′ to C-7′ (*δ*_C_ 153.2), C-8′ (*δ*_C_ 139.7), C-9′ (*δ*_C_ 195.4), H_3_-4″ to C-2″ (*δ*_C_ 120.5), C-3″ (*δ*_C_ 137.7), C-5″ (*δ*_C_ 18.2), and the COSY correlations (Figure 2) of H_2_-1′/H-2′, H_2_-6′/H-7′, and H_2_-1″/H-2″ (*δ*_H_ 5.30). In addition, the COSY correlation of H_2_-6′/H-2′ indicated that the C-6′ of one prenyl unit was connected to another prenyl unit at C-2′, forming a geranyl unit. An aldehyde group at C-9′ and a terminal double bond at C-3′ and C-4′ were further demonstrated by the HMBC correlations of –CHO (*δ*_H_ 9.33, s) to C-8′, C-10′ (*δ*_C_ 9.5) and H_2_-4′ (*δ*_H_ 4.78 and 4.70, both s) to C-2′, C-5′ (*δ*_C_ 19.4). The geranyl and the prenyl units were determined to be located at the C-3 and C-5 of *p*-hydroxybenzaldehyde, respectively, from the HMBC correlations of H_2_-1′ to C-2 (*δ*_C_ 131.4), C-3 (*δ*_C_ 127.9), C-4 (*δ*_C_ 158.9), H_2_-1″ to C-4, C-5 (*δ*_C_ 126.6), C-6 (*δ*_C_ 130.7), and –CHO (*δ*_H_ 9.82, s) to C-1 (*δ*_C_ 129.5), C-2, C-6. In summary, the planar structure of **5** was depicted as shown (Figure 1).

The absolute configuration of C-2′ was determined by the ECD calculation. The results (Figure 3) shown that the calculated ECD spectrum of (2′*S*)-**5** agrees well with the experimental one, which allowed the absolute configuration of **5** to be specified as 2′*S*. As a result, the structure of claulenin B (**5**) was clarified.

Thirty-two known compounds were identified as 4-methoxy-*N*-methyl-2-quinolone (**6**) [29], (–)-(*S*)-edulinine (**7**) [30], dictamine (**8**) [31], pteleine (**9**) [32], cinnamamide (**10**) [33], *N*-methylcinnamamide (**11**) [34], (*Z*)-*N*-methyl-3-phenylacrylamide (**12**) [35], *N*-2-phenylethylcinnamamide (**13**) [36], lansiumamide A (**14**) [37], lansiumamide B (**15**) [37], (2*E*)-3-phenyl-*N*-[(*E*)-2-phenylvinyl]acrylamide (**16**) [38], lansamide I (**17**) [39], 2-benzothiazolol (**18**) [40], indole (**19**) [41], *N*,*N′*-bis[2(1*H*-indol-3-yl)ethyl]urea (**20**) [42], demethylsuberosin (**21**) [43], swietenocoumarin I (**22**) [44], xanthyletin (**23**) [45], 3’-hydroxyxanthyletn (**24**) [46], (–)-3-(*R*)-decursinol (**25**) [47], dimethyl allyl xanthyletin (**26**) [48], 3-(1,1-dimethylallyl)decursinol (**27**) [49,50], imperatorin (**28**) [51], phellopterin (**29**) [52], (–)-heraclenol (**30**) [53,54], (+)-isoangenomalin (**31**) [55], nodakenetin (**32**) [56], chalepin (**33**) [57], 6-methoxymicrominutinin (**34**) [58], marmesinin (**35**) [59], marmesin glycoside (**36**) [60], (2*R*)-2′-hydroxymarmesin (**37**) [61], and (4′-methyl-[1,1′-biphenyl]-2-yl)(*p*-tolyl)methanone (**38**) [62], by comparing their spectroscopic data with those reported in the literature. The absolute configuration of **22** was first defined as *S* by comparing the calculated and experimental ECD spectra (Figure S51, Supporting Information). In addition, **20** and **38** were reported as natural products for the first time, and **7**–**10**, **12**, **16**, **18**–**21**, **24**, **25**, **27**, **29**, **32**, and **34**–**38** and all of the isolates except **23**, **26**, **28**, **31**, and **33** were obtained for the first time from *Clausena* species and *C. lenis*, respectively.

### 2.2. Anti-Neuroinflammatory Activities

**Figure 5:**
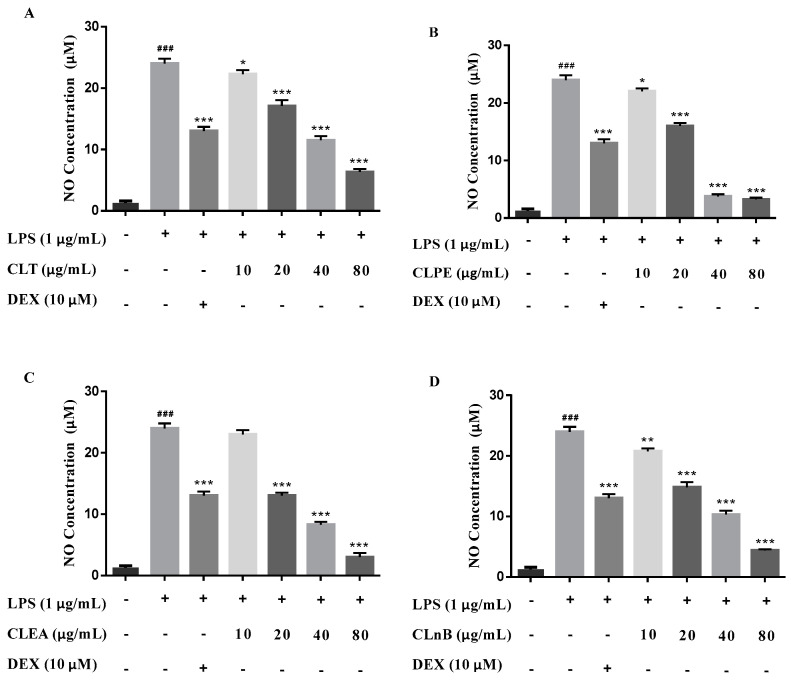
The production of NO stimulated by LPS in BV-2 cells. (**A**) Inhibitory effects of CLT on LPS-induced NO production in BV-2 cells. (**B**) Inhibitory effects of CLPE on LPS-induced NO production in BV-2 cells. (**C**) Inhibitory effects of CLEA on LPS-induced NO production in BV-2 cells. (**D**) Inhibitory effects of CLnB on LPS-induced NO production in BV-2 cells. Data were presented as the mean ± SD of three independent experiments. ^###^ *p* < 0.001 *versus* control group, * *p* < 0.05, ** *p* < 0.01, *** *p* < 0.001 *versus* LPS group. (CLT: the 95% and 50% ethanol total extract of *C. lenis*; CLPE: petroleum ether extract of *C. lenis*; CLEA: ethyl acetate extract of *C. lenis*; CLnB: *n*-BuOH extract of *C. lenis*; DEX: dexamethasone, as the positive control).

The 95% and 50% ethanol extract (CLT) of *C. lenis*, along with its three partitioned extracts with different polarity solvents, i.e., petroleum ether extract (CLPE), ethyl acetate extract (CLEA), and *n*-BuOH extract (CLnB) were evaluated for anti-neuroinflammatory activities based on reduction of NO production stimulated by LPS in BV-2 microglial cells. The results exhibited that NO production can be dose-dependently inhibited in the range of 10–80 μg/mL (Figure 5). To further investigate which compounds are responsible for the effect, all of these isolates (**1**–**38**) were subjected to an evaluation of their anti-neuroinflammatory activities using the same method. Six compounds (**2**, **23**, **27**, **28**, **33**, and **34**) inhibited NO production by more than 50% at 50 μM and their IC_50_ values were finally determined to be from 17.6 to 40.9 μM (Table 2). Dexamethasone (DEX) was used as a positive control. Alongside this, no cytotoxicity was observed in BV-2 microglial cells treated by these test subjects at 50 μM (cell viability > 95%).

### 2.3. Interactions of Bioactive Compounds with iNOS

**Figure 6:**
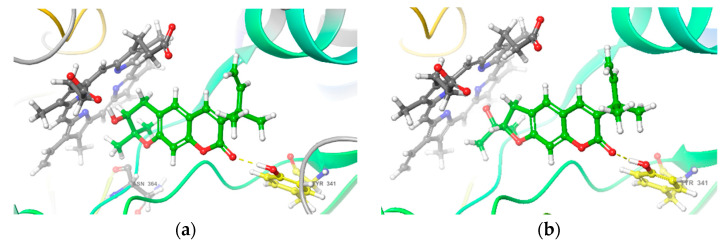
The docking view of interaction between iNOS and compounds **27** (**a**) or **33** (**b**).

Nitric oxide synthases (NOSs) catalyze the NO production using oxygen and nitrogen derived from arginine. Inducible NOS (iNOS) is one of the isoenzymes of NOSs and plays a major part in NO production during inflammation [63]. In recent years, structure-based calculations have been widely used to predict the pharmacological mechanisms of active compounds, among which molecular docking is a commonly used method [64]. To explore the possible mechanism of those bioactive compounds against NO production, we investigated the interaction between iNOS and compounds **2**, **23**, **27**, **28**, **33**, and **34** by molecular docking [65,66]. The results showed that compounds **27** and **33** had good affinities with iNOS (Glide Score < −5) (Table 3), and both of them have a hydrogen bond with the residues TYR341 of iNOS (Figure 6). Therefore, the possible mechanism of NO inhibition of **27** and **33** is through interaction with iNOS by targeting the residues in the active pocket of iNOS.

## 3. Discussion

Thirty-seven compounds were isolated and identified from *C. lenis* and five of them are new ones (**1**–**5**). The structures were elucidated based on MS, UV, IR, and NMR spectroscopic data and comparison with the data reported in literature. The absolute configurations of new compounds were characterized by using the ECD calculations and single-crystal X-ray diffraction analysis. (±)-Claulenine A (**1**) is a chlorine-containing furoquinoline racemate. Chlorinated natural products are rare in terrestrial plants, but common in bacteria, marine animals, and macroalgae [67,68]. **1** could be an artifact produced in the isolation procedure, but could also be generated by Fe(II)- and 2-oxoglutarate-dependent halogenases (2ODHs) [69]. The LC/MS analysis of the fresh *C. lenis* proved **1** to be originated from a natural source, but the real chlorine source is not clear. Claulenine B (**2**) is a high conjugated amide alkaloid. Claulenin A (**3**) is a prenylated coumarin with an oxirane ring and clauleside A (**4**) is a furocoumarin glucoside. Claulenin B (**5**) is a *p*-hydroxybenzaldehyde with multi-prenyl substituents. Of the known compounds, **20** and **38** are the first reported natural products, and 20 and 28 compounds are obtained from *Clausena* species and *C. lenis*, respectively, for the first time. Most of these compounds are prenylated, so the prenyltransferases play an important role in their biosyntheses.

The ethanol extract and its partitioned extracts with different polarities from *C. lenis* have significant inhibitory effects on NO production in LPS-induced BV-2 microglial cells. In the subsequent bioactivity-guided fractionation, compounds **2**, **23**, **27**, **28**, **33**, and **34** were disclosed to be the potentially active compounds with inhibition effects. Most of the bioactive compounds are coumarins, suggesting that coumarins might be the main bioactive substances for the anti-neuroinflammatory properties of the extract of *C. lenis*. Furthermore, the molecular docking results revealed that **27** and **33** had a good interaction with iNOS, which could be one of the mechanisms for their anti-inflammation effects.

## 4. Materials and Methods

### 4.1. General Experimental Procedures

Optical rotations were measured on a Rudolph Autopol IV automatic polarimeter (Rudolph Research Analytical, Hackettstown, NJ, USA). The electronic circular dichroism (ECD) and UV data were acquired on a JASCO, J-1500 CD spectrophotometer (JASCO, Tokyo, Japan). IR spectra were recorded on a Thermo Scientific Nicolet iS50 FT-IR spectrometer (Thermo Fisher Scientific, Waltham, MA, USA). HR-ESI-MS data were measured on a Waters Xevo G2 Q-TOF mass spectrometer (Waters Co., Milford, MA, USA). The nuclear magnetic resonance (NMR) spectra were obtained through a Varian INOVA-500 NMR spectrometer (Varian Co., Palo Alto, CA, USA) with TMS as an internal standard. The column chromatography (CC) was undertaken on silica gel (100−200 and 200−300 mesh, Qingdao Marine Chemical Co., Ltd., Qingdao, China), ODS-A-HG (50 µm; YMC Co., Ltd., Kyoto, Japan), and Sephadex LH-20 (Amersham Pharmacia, Uppsala, Sweden). Preparative TLC and TLC analyses were carried out on the pre-coated silica gel GF254 plates (Qingdao Marine Chemical Co., Ltd., Qingdao, China). Semi-preparative HPLC was carried out using an Agilent Eclipse XDB-C18 column (9.4 mm × 250 mm, i.d., 5 μm) on an Agilent 1260 series LC instrument with a DAD detector (Agilent Technologies, Santa Clara, CA, USA).

### 4.2. Plant Material

In November 2019, dry leaves and stems of *Clausena lenis* Drake were collected from Baoting County, Hainan Province, People’s Republic of China. Botanical identification was made by Prof. Pengfei Tu, one of the authors, and a voucher specimen (No. CL201911) was kept in the herbarium of Modern Research Center for Traditional Chinese Medicine, Peking University.

### 4.3. Extraction and Isolation

The air-dried leaves and stems of *C.* *lenis* (20.0 kg) were refluxed with 95% and 50% aqueous ethanol (160 L × 2 h × 2), respectively, and concentrated under reduced pressure to obtain 1.8 kg dry total extract (CLT). The extract was suspended in H_2_O and extracted with petroleum ether (CLPE), ethyl acetate (CLEA), and *n*-BuOH (CLnB), successively.

The petroleum ether extract (179.1 g) was eluted by gradient elution of petroleum ether-ethyl acetate (1:0, 50:1, 30:1, 20:1, 10:1, 5:1, 3:1, 1:1, and 0:1, *v/v*) on a silica gel column to obtain 14 fractions (A−N). Fraction J (1.83 g) was subjected to Sephadex LH-20 (MeOH–CH_2_Cl_2_, 1:1, *v*/*v*) and produced five subfractions (J.1−J.5). Subfractions J.3 (0.70 g) and J.4 (0.27 g) were purified by semi-preparative HPLC (3.0 mL/min) to yield **2** (4.2 mg, 47% aqueous acetonitrile, *t*_R_ 29.2 min) and **9** (3.8 mg, 40% aqueous acetonitrile, *t*_R_ 15.6 min), respectively. Fraction K (14.2 g) was divided into seven fractions (K.1–K.7) by a ODS-A-HG column (aqueous MeOH, 30%–100%). Compounds **33** (26.6 mg, *t*_R_ 36.7 min) and **27** (4.5 mg, *t*_R_ 53.8 min) were obtained from subfraction K.4 (780 mg) after purification by semi-preparative HPLC (3.0 mL/min, 42% aqueous acetonitrile). Similarly, **34** (2.8 mg, *t*_R_ 16.9 min) was purified from subfraction K.2 (50 mg) by semi-preparative HPLC (3.0 mL/min, 35% aqueous acetonitrile). Fraction L (3.42 g) was fractionated into four parts (L.1–L.4) by gel filtration on Sephadex LH-20 (MeOH–CH_2_Cl_2_, 1:1, *v*/*v*). Subfraction L.2 (1.85 g) was subjected to an ODS-A-HG column (aqueous MeOH, 25%–100%) to give eight fractions (L.2.1–L.2.8). Subfractions L.2.1 (121.2 mg) and L.2.5 (83.4 mg) were further purified by semi-preparative HPLC (3.0 mL/min) to yield **12** (3.2 mg, 15% aqueous acetonitrile, *t*_R_ 21.8 min) and **22** (6.0 mg, 35% aqueous acetonitrile, *t*_R_ 39.9 min), respectively. Likewise, subfraction L.2.3 (131.4 mg) was purified to yield **32** (27.2 mg, 25% aqueous acetonitrile, *t*_R_ 15.7 min) and **25** (3.8 mg, 25% aqueous acetonitrile, *t*_R_ 18.4 min).

The ethyl acetate extract (160.9 g) was chromatographed on silica gel eluted with petroleum ether-ethyl acetate (1:0, 50:1, 30:1, 20:1, 10:1, 5:1, 3:1, 1:1, and 0:1, *v*/*v*) to afford 15 fractions (A−O). Fraction C (0.29 g) was fractionated on a Sephadex LH-20 column (MeOH–CH_2_Cl_2_, 1:1, *v*/*v*) to yield five parts (C.1–C.5). Subfraction C.3 (89.4 mg) was purified by semi-preparative HPLC (3.0 mL/min, 60% aqueous acetonitrile) to obtain **38** (1.1 mg, *t*_R_ 44.8 min) and **26** (5.1 mg, *t*_R_ 48.5 min). Fraction F (9.63 g) was divided into five parts (F.1–F.5) by an MCI GEL CHP20 column (aqueous MeOH, 50%–100%). Subfraction F.1 (129 mg) was purified by semi-preparative HPLC (3.0 mL/min, 28% aqueous acetonitrile) to yield **10** (2.7 mg, *t*_R_ 12.7 min) and **11** (17.0 mg, *t*_R_ 14.9 min). Subfraction F.3 (7.09 g) was applied to an ODS-A-HG column (aqueous MeOH, 10%–100%) and further purified by preparative HPLC (3.0 mL/min, 60% aqueous acetonitrile) to yield **14** (6.3 mg, *t*_R_ 14.4 min) and **17** (143.5 mg, *t*_R_ 21.4 min). Fraction G (7.6 g) was submitted to Sephadex LH-20 (MeOH) to acquire seven portions (G.1–G.7). Subfraction G.4 (92.3 mg) was purified by semi-preparative HPLC (50% aqueous acetonitrile, 3 mL/min) to yield **23** (2.2 mg, *t*_R_ 15.8 mind). Fraction H (4.27 g) was subjected to Sephadex LH-20 (MeOH–CH_2_Cl_2_, 1:1, *v*/*v*) to afford three fractions. Subfraction H.3 (130 mg) was purified by semi-preparative HPLC (3.0 mL/min, 50% aqueous acetonitrile) to yield **15** (2.0 mg, *t*_R_ 24.9 min). Fraction I (2.88 g) was separated into five fractions (I.1−I.5) using Sephadex LH-20 (MeOH). Subfraction I.3 (318 mg) was purified by semi-preparative HPLC (3.0 mL/min, 50% aqueous acetonitrile) to yield **31** (3.3 mg, *t*_R_ 13.8 min), **28** (3.4 mg, *t*_R_ 21.2 min), **29** (6.7 mg, *t*_R_ 25.2 min), and **5** (7.8 mg, *t*_R_ 50.5 min). In the same way, **8** (5.5 mg, 60% aqueous acetonitrile, *t*_R_ 7.1 min) and **16** (6.1 mg, 60% aqueous acetonitrile, *t*_R_ 10.0 min) were obtained from subfraction I.4 (325 mg). Subfraction I.5 (0.93 g) was applied to an ODS-A-HG column (aqueous MeOH, 50%–100%) and further purified by preparative HPLC (3.0 mL/min, 25% aqueous acetonitrile) to yield **18** (8.5 mg, *t*_R_ 17.8 min). Fraction K (16.5 g) was fractionated by Sephadex LH-20 (MeOH–CH_2_Cl_2_, 1:1, *v*/*v*) to give five fractions. Subfraction K.3 (0.75 g) was separated into five fractions by an ODS-A-HG column (aqueous MeOH, 20%–100%). Subfraction K.3.2 (140 mg) was subjected to preparative TLC (petroleum ether-ethyl acetate, 3:1, *v*/*v*) and further purified by semi-preparative HPLC (3 mL/min, 22% aqueous acetonitrile) to afford **21** (1.9 mg, *t*_R_ = 46.2 min). Fraction L (2.86 g) was subjected to Sephadex LH-20 (MeOH–CH_2_Cl_2_, 1:1, *v*/*v*) to afford five fractions. Subfraction L.3 (0.56 g) and L.4 (0.30 g) was submitted to an ODS-A-HG column (aqueous MeOH, 30%–100%) to yield eight and six fractions, respectively. And subfractions L.3.4 (107.6 mg) and L.4.3 (71.3 mg) were purified by semi-preparative HPLC (3.0 mL/min) to yield **13** (3.1 mg, *t*_R_ 13.5 min, 50% aqueous acetonitrile) and **20** (0.5 mg, *t*_R_ 30.6 min, 35% aqueous acetonitrile), respectively. Fraction M (17.1 g) was applied to a silica gel column, eluting with petroleum ether-ethyl acetate (10:1, 8:1, 5:1, 3:1, and 1:1, *v*/*v*) to obtain fractions from M.1 to M.8. Subfraction M.5 (1.7 g) was subjected to Sephadex LH-20 (MeOH–CH_2_Cl_2_, 1:1, *v*/*v*) to afford three fractions. Subfraction M.5.2 (1.26 g) was submitted to an ODS-A-HG column (aqueous MeOH, 10%–100%) to yield three fractions. Among them, subfraction M.5.2.2 (135.1 mg) was purified by semi-preparative HPLC (3.0 mL/min, 45% aqueous acetonitrile) to yield **3** (3.8 mg, *t*_R_ 28.5 min).

The *n*-BuOH extract (222.8 g) was subjected to macroporous resin HP-20 (aqueous ethanol, 0%, 20%, 50%, 70%, and 95%) to provide five portions (A–E). Fraction C (27.3 g) was divided into nine parts (C.1–C.9) by an ODS-A-HG column (aqueous MeOH, 5%–100%). Subfraction C.4 (2.50 g) was separated into three fractions (C.4.1–C.4.3) using a Sephadex LH-20 column (MeOH–CH_2_Cl_2_, 1:1, *v*/*v*). Subfraction C.4.2 (1.44 g) was subjected to a silica gel column with a stepwise gradient of CH_2_Cl_2_–MeOH (20:1, 15:1, 10:1, 8:1, 5:1, 3:1, and 1:1) to produce nine fractions (C.4.2.1−C.4.2.9). Subfractions C.4.2.6 (210 mg) were purified by semi-preparative HPLC (3.0 mL/min) to yield **37** (2.8 mg, 15% aqueous acetonitrile, *t*_R_ 12.8 min). Subfraction C.5 (2.77 g) was separated by a Sephadex LH-20 column (MeOH–CH_2_Cl_2_, 1:1, *v/v*) to obtain three fractions (C.5.1–C.5.3). Subfraction C.5.2 (1.64 g) was further purified by a silica gel column with a gradient of CH_2_Cl_2_–MeOH system (20:1, 10:1, 8:1, 5:1, 1:1, and 0:1) to get **35** (9.8 mg). In addition, subfraction C.5.3 (180 mg) was further purified by preparative TLC (CH_2_Cl_2_–MeOH, 8:1, *v*/*v*) and semi-preparative HPLC (3.0 mL/min, 20% aqueous acetonitrile) to yield **36** (1.8 mg, *t*_R_ 27.3 min). Subfraction C.7 (2.36 g) was fractionated into four parts (C.7.1–C.7.4) by using Sephadex LH-20 (MeOH–CH_2_Cl_2_, 1:1, *v*/*v*). Subfraction C.7.2 (1.29 g) was eluted by gradient elution of CH_2_Cl_2_–MeOH (1:0, 100:1, 50:1, 20:1, 10:1, 8:1, 5:1, and 1:1, *v*/*v*) on a silica gel column to obtain nine fractions (C.7.2.1− C.7.2.9). Subfractions C.7.2.3 (13.5 mg) and C.7.2.5 (83.2 mg) were purified by semi-preparative HPLC (3.0 mL/min) to yield **24** (0.6 mg, 25% aqueous acetonitrile, *t*_R_ 23.4 min) and **7** (3.2 mg, 20% aqueous acetonitrile, *t*_R_ 37.8 min). Fraction D (30.5 g) was divided into eight parts (D.1–D.8) by an ODS-A-HG column (aqueous MeOH, 10%–100%). Subfraction D.4 (1.9 g) was fractionated into three parts (D.4.1–D.4.3) by using Sephadex LH-20 (MeOH–CH_2_Cl_2_, 1:1, *v*/*v*). Subfraction D.4.2 (1.03 g) was applied to a silica gel column (CH_2_Cl_2_–MeOH, 10:1, 5:1, 1:1, and 0:1, *v*/*v)* to produce seven subfractions (D.4.2.1−D.4.2.7). Purification of subfraction D.4.2.4 (130 mg) by semi-preparative HPLC with 16% aqueous acetonitrile (3.0 mL/min) afforded **4** (22.7 mg, *t*_R_ 22.8 min). Subfraction D.5 (2.6 g) was subjected to a Sephadex LH-20 column (MeOH–CH_2_Cl_2_, 1:1, *v*/*v*) to afford three fractions (D.5.1−D.5.3). Subfraction D.5.2 (1.07 g) was applied to a silica gel column, eluting with CH_2_Cl_2_–MeOH (100:1, 50:1, 20:1, 10:1, 5:1, 1:1, and 0:1, *v*/*v*), to give seven fractions (D5.2.1−D.5.2.7). Subfraction D.5.2.1 (90 mg) was further purified by semi-preparative HPLC (3.0 mL/min, 18% aqueous acetonitrile) to afford **30** (1.0 mg, *t*_R_ 45.4 min). Fraction E (3.3 g) was fractionated into five parts (E.1–E.5) by gel filtration on Sephadex LH-20 (MeOH–CH_2_Cl_2_, 1:1, *v*/*v*). Subfraction E.2 (1.72 g) was subjected to a silica gel column (petroleum ether–ethyl acetate, 5:1, 3:1, 2:1, 1:1, and 0:1, *v*/*v*) to give nine fractions (E.2.1–E.2.9). Subfraction E.2.8 (245.1 mg) was further purified by semi-preparative HPLC (3.0 mL/min, 50% aqueous acetonitrile) to yield **6** (1.6 mg, *t*_R_ 9.0 min) and **1** (4.1 mg, *t*_R_ 17.1 min). The chiral HPLC separation of racemic **1** was performed using a Chiralpak IA column (10 × 250 mm, 5 mm, Daicel, Nanning, China), eluting with *n*-hexane-isopropanol (85:15, *v/v*). The detection wavelength was 254 nm, the column temperature was 30 °C, and the flow rate was 3 mL/min. Finally, compounds (+)-**1a** (1.9 mg, *t*_R_ 18.7 min) and (−)-**1b** (1.8 mg, *t*_R_ 22.7 min) were obtained. Subfraction E.3 (0.49 g) was subjected to a silica gel column (petroleum ether–ethyl acetate, 1:0, 50:1, 30:1, 10:1, 5:1, 3:1, and 1:1, *v/v*) to give **19** (5.5 mg).

#### 4.3.1. (±)-Claulenine A (**1**)

White solid; ${\left[\alpha \right]}_{D}^{25}$ 0 (*c* 0.12, MeOH); UV (MeOH) *λ*_max_ (log *ε*) 337 (3.54), 321 (3.82), 309 (3.68), 247 (4.46) nm; IR (KBr) *ν*_max_ 2932, 1622, 1589, 1507, 1482, 1433, 1368, 1260, 1215, 1089, 1011 cm^−1^; ^1^H and ^13^C-NMR data, see Table 1; HR-ESI-MS *m/z* 366.1101 [M + H]^+^ (calcd for C_18_H_21_NO_5_Cl, 366.1108).

*(+)-Claulenine A* [(+)-**1a**]: White solid, ${\left[\alpha \right]}_{D}^{25}$ + 38 (*c* 0.12, MeOH); ECD (MeOH) *λ*_max_ (Δ*ε*) 246 (−2.00), 238 (+1.62), 222 (−3.61) nm.

(*−)-Claulenine A* [(−)-**1b**]: White solid, ${\left[\alpha \right]}_{D}^{25}$ − 42 (*c* 0.10, MeOH); ECD (MeOH) *λ*_max_ (Δ*ε*) 247 (+2.44), 238 (−1.41), 223 (+2.99) nm.

#### 4.3.2. Claulenine B (**2**)

Yellow oil; UV (MeOH) *λ*_max_ (log *ε*) 290 (3.52), 267 (3.57), 219 (3.54), 200 (3.72) nm; IR (KBr) *ν*_max_ 2932, 1653, 1607, 1510, 1451, 1371, 1253, 1172, 1086, 1032, 765 cm^−1^; ^1^H and ^13^C-NMR data, see Table 1; HR-ESI-MS *m*/*z* 294.1490 [M + H]^+^ (calcd for C_19_H_20_NO_2_, 294.1494).

#### 4.3.3. Claulenin A (**3**)

Yellow oil; ${\left[\alpha \right]}_{D}^{25}$ − 25 (*c* 0.24, MeOH); ECD (MeOH) *λ*_max_ (Δ*ε*) 232 (+3.12), 222 (+2.37), 206 (+11.31) nm; UV (MeOH) *λ*_max_ (log *ε*) 335 (3.20), 298 (2.89), 250 (3.34), 227 (3.26), 202 (3.68) nm; IR (KBr) *ν*_max_ 2967, 1715, 1626, 1581, 1490, 1268, 1164, 1131, 986, 784 cm^−1^; ^1^H and ^13^C-NMR data, see Table 1; HR-ESI-MS *m*/*z* 315.1594 [M + H]^+^ (calcd for C_19_H_23_O_4_, 315.1596).

#### 4.3.4. Clauleside A (**4**)

Yellow solid; ${\left[\alpha \right]}_{D}^{25}$ + 27 (*c* 0.32, MeOH); ECD (MeOH) *λ*_max_ (Δ*ε*) 336 (+10.20), 270 (+1.55), 252 (+8.75), 236 (+4.69), 229 (+9.56), 211 (−3.48) nm; UV (MeOH) *λ*_max_ (log *ε*) 334 (3.93), 250 (3.79), 226 (3.94), 206 (4.20) nm; IR (KBr) *ν*_max_ 3381, 2932, 2882, 1712, 1626, 1568, 1486, 1446, 1363, 1124, 1076, 1038, 823, 610 cm^−1^; ^1^H and ^13^C-NMR data, see Table 1; HR-ESI-MS *m*/*z* 405.1187 [M − H]^−^ (calcd for C_20_H_21_O_9_, 405.1186).

#### 4.3.5. Claulenin B (**5**)

Yellow oil; ${\left[\alpha \right]}_{D}^{25}$ − 10 (*c* 0.20, MeOH); ECD (MeOH) *λ*_max_ (Δ*ε*) 244 (−2.18), 219 (+1.40) nm; UV (MeOH) *λ*_max_ (log *ε*) 282 (3.24), 229 (3.61), 211 (3.52) nm; IR (KBr) *ν*_max_ 3421, 2935, 1687, 1602, 1442, 1378, 1291, 1125, 977, 896 cm^−1^; ^1^H and ^13^C NMR data, see Table 1; HR-ESI-MS *m*/*z* 363.1933 [M + Na]^+^ (calcd for C_22_H_28_O_3_Na, 363.1936).

#### 4.3.6. (*S*)-Swietenocoumarin I (**22**)

Colorless oil; ${\left[\alpha \right]}_{D}^{25}$ + 30 (*c* 0.20, MeOH); ECD (MeOH) *λ*_max_ (Δ*ε*) 226 (+5.80), 204 (−12.02) nm; ESI-MS *m*/*z* 347 [M + H]^+^.

### 4.4. ECD Calculations of ***1***, ***3***, ***4a***, ***5***, and ***22***

Conformation searches of **1**, **3**, **4a**, **5**, and **22** using molecular mechanics calculations were performed in Spartan 14 (Wavefunction Ind.) with MMFF force field with an energy window for acceptable conformers (ewindow) of 5 kcal/mol above the ground state. Then the predominant conformers were optimized by using the TDDFT method at B3LYP/6-31G(d) level in Gaussian 16 [70]. The optimized conformers were used for the ECD calculations, which were performed with Gaussian 16 at B3LYP/6-31+G(d) level. The solvent effects were taken into account by the polarizable-conductor calculation model (PCM, methanol as the solvent). The SpecDis v1.71 program was used to generate ECD calculation curves [71].

### 4.5. X-ray Crystallography of (+)-***1a***

The single crystals of (+)-**1a** were collected from methanol solution at room temperature. An Agilent Gemini E X-ray single-crystal diffractometer with an Oxford Cryostream cooler was used to collect the single crystal data with Cu Kα radiation at T = 174.7 K. The structure was solved with the direct method using SHELXS-97 and refined anisotropically by full-matrix least-squares on F2 using SHELXL-97. The H atoms were placed in calculated positions and refined using a riding model. The absolute configuration was determined by refinement of the Flack parameter based on resonant scattering of the light atoms.

Crystal data for (+)-**1a**: C_18_H_20_ClNO_5_ (M = 365.80 g/mol), monoclinic, space group P2_1_, size 0.19 × 0.17 × 0.02 mm^3^, a = 11.1628(2) Å, b = 6.63600(10) Å, c = 11.6688(2) Å, α = 90°, β = 97.049(2)°, γ = 90°, V = 857.85(3) Å^3^, Z = 2, T = 100.00(10) K, μ(Cu Kα) = 2.230 mm^−1^, 3400 unique (R_int_ = 0.0736, R_sigma_ = 0.0267), which were used in all calculations. The final R_1_ was 0.0384 [I > 2σ(I)] and the final wR_2_ was 0.1060 (all data). Flack parameter −0.003(9). Crystallographic data for **1** have been filed with Cambridge Crystallographic Data Centre (CCDC, deposition number: CCDC 2129405). These data can be obtained free of charge from the CCDC via https://www.ccdc.cam.ac.uk/structures/ (accessed on 17 December 2021).

### 4.6. Absolute Configurations Determination of Sugar Moiety for ***4***

Compound **4** (1.0 mg) was hydrolyzed with 2 mol/L HCl (5 mL) for 5 h at 85 °C. The reaction product was extracted three times with CH_2_Cl_2_. After the aqueous layer was concentrated to dryness, 2 mL anhydrous pyridine containing 2.0 mg L-cysteine methyl ester hydrochloride was added and heated at 60 °C for 1 h. Subsequently, o-tolylisothiocyanate (10 μL) was added and heated at 60 °C for 1 h. D-glucose standard (2 mg) and L-glucose standard (2 mg) were respectively reacted in the same procedure. Then, each reaction mixture was filtered by a 0.22 μm membrane and analyzed directly by an Agilent Extended C18 column (250 mm × 4.6 mm, 5 μm) on an Agilent 1260 HPLC with a gradient elution of MeCN–H_2_O (5:95–30:70, *v*/*v*, 0–30 min, 1.0 mL/min) at 30 °C, and UV detection wavelength was 210 nm (Figure S40, Supporting Information). The sugar moiety of **4** was detected as D-glucose by the same t_R_ value with that of the D-glucose standard derivative [72].

### 4.7. Cell Culture

Mouse BV-2 microglial cells (Peking Union Medical College Cell Bank, Beijing, China) were cultured in Dulbecco’s Modified Eagle’s Medium (Macgene, Beijing, China) and supplemented with 10% fetal bovine serum (Gibico, Waltham, MA, USA), penicillin (Macgene, 100 U/mL, Beijing, China), and streptomycin (Macgene, 100 μg/mL) in a humidified incubator containing 95% air and 5% CO_2_ at 37 °C.

### 4.8. Nitric Oxide (NO) Production Measurement and Cell Viability Assay

BV-2 cells (1 × 10^5^ cells/well) were cultured in 48-well plates and stimulated with 1.0 μg/mL LPS (*Escherichia coli* 0111:B4, Sigma, St. Louis, MO, USA) with or without test extracts or compounds at 37 °C for 24 h. The production of NO was tested using a commercial assay kit (Nanjing Jiancheng Bioengineering Institute, Nanjing, Jiangsu, China), according to the manufacturer’s instructions. Cell culture supernatants (160 μL) were reacted with 80 μL of the Griess reagent (1% sulfanilamide, 0.1% naphthylethylene diaminedihydrochloride, and 2% phosphoric acid) for 10 min in the dark at room temperature. The absorbance was measured at 540 nm using a microplate reader (Tecan Trading AG, Basel, Switzerland). The experiments were performed in triplicate, and the results are presented as the mean ± SD of three independent experiments. The cell viability was evaluated according to MTT assay. Dexamethasone was used as the positive control.

### 4.9. Molecular Docking

The crystal structure of iNOS (PDB ID: 3E6T) was obtained from the Protein Data Bank of RCSB (Research Collaboratory for Structural Bioinformatics). Docking simulations between bioactive compounds and iNOS were performed using the Maestro software suite 2015 (Schrodinger, New York, NY, USA). The ligand molecules were drawn with Chem3D Pro 14.0 (CambridgeSoft, Waltham, MA, USA) and optimized by the Ligprep module of Maestro. The protein receptor was prepared by deleting the ligand and water molecules and then was adopted for molecular docking with ligands. The reported inhibitor binding sites of iNOS was chosen as the binding pocket [73].

## Figures and Tables

**Table 1:** ^1^H-NMR (500 MHz) and ^13^C (125 MHz) NMR Data of **1**–**5** (*δ* in ppm).

No.	1 ^a^		2 ^a^		3 ^a^		4 ^b^		5 ^a^	
	*δ*_H_ (*J* in Hz)	*δ_C_*, Type	*δ*_H_ (*J* in Hz)	*δ_C_*, Type	*δ*_H_ (*J* in Hz)	*δ_C_*, Type	*δ*_H_ (*J* in Hz)	*δ_C_*, Type	*δ*_H_ (*J* in Hz)	*δ_C_*, Type
1				135.4, C						129.5, C
2	7.59, d (2.7)	143.0, CH	7.33, overlap	128.8, CH		160.4, C		163.6, C	7.52, br s	131.4, CH
3	7.00, d (2.7)	104.5, CH	7.45, m	128.1, CH		131.0, C	6.13, d (9.4)	112.4, CH		127.9, C
3a		103.0, C								
4		154.4, C	7.33, overlap	129.8, CH	7.47, s	138.2, CH	7.77, d (9.4)	146.2, CH		158.9, C
4a		113.2, C				113.3, C		114.3, C		
5	7.45, s	100.1, CH	7.45, m	128.1, CH	7.19, s	123.4, CH	7.33, s	125.3, CH		126.6, C
6		148.1, C	7.33, overlap	128.8, CH		124.7, C		126.7, C	7.52, br s	130.7, CH
7		152.8, C	7.64, d (15.5)	142.7, CH		162.4, C		164.7, C	9.82, s	191.4, CH
8	7.34, s	106.8, CH	6.95, d (15.5)	118.5, CH	6.70, s	97.3, CH	6.67, s	98.3, CH		
8a		142.7, C				154.8, C		156.9, C		
9		162.9, C		166.7, C						
1′	4.93, dd (9.8, 3.7)	72.5, CH_2_	6.38, d (8.6)	127.1, CH		40.4, C	3.43, dd (15.9, 9.5)	34.7, CH_2_	2.78, d (7.4, 3.8)	34.7, CH_2_
	4.75, dd (9.8, 6.8)						3.16, overlap			
2′	4.18, dd (6.8, 3.7)	77.1, CH	6.20, d (8.6)	125.6, CH	6.16, dd (17.4, 10.7)	145.7, CH	5.47, t-like (8.7)	86.3, CH	2.68, m	46.4, CH
3′		71.9, C		127.0, C	5.08, d (10.7)	112.2, CH_2_		145.6, C		146.2, C
					5.07, d (17.4)					
4′	1.77, s	29.5, CH_3_	7.28, d (8.7)	130.2, CH	1.46, s	24.2, CH_3_	4.44, d (12.8)	70.0, CH_2_	4.78, s	112.8, CH_2_
							4.19, d (12.8)		4.70, s	
5′	1.77, s	29.0, CH_3_	6.83, d (8.7)	114.2, CH	1.35, s	24.2, CH_3_	5.26, d (13.9)	114.1, CH_2_	1.71, s	19.4, CH_3_
6′	4.00, s	56.1, CH_3_		159.5, C					2.44, m	32.3, CH_2_
7′	4.03, s	56.2, CH_3_	6.83, d (8.7)	114.2, CH					6.35, t (7.2)	153.2, CH
8′			7.28, d (8.7)	130.2, CH						139.7, C
9′			3.78, s	55.4, CH_3_					9.33, s	195.4, CH
10′			3.10, s	34.6, CH_3_					1.70, s	9.5, CH_3_
1″					3.19, m	29.7, CH_2_	4.23, d (7.8)	103.6, CH	3.43, d (7.2)	30.7, CH_2_
2″					4.71, t (8.8)	91.0, CH	3.16, overlap	75.0, CH	5.30, m	120.5, CH
3″						71.8, C	3.38, m	78.0, CH		137.7, C
4″					1.46, s	26.2, CH_3_	3.21, overlap	71.6, CH	1.82, s	26.0, CH_3_
5″					1.22, s	24.4, CH_3_	3.21, overlap	78.0, CH	1.84, s	18.2, CH_3_
6″							3.81, d (11.9)	62.7, CH_2_		
							3.60, dd (11.9, 4.9)			

^a^ measured in CDCl_3_. ^b^ measured in MeOD.

**Table 2:** NO inhibition toward LPS-induced BV-2 cells.

Compound	IC_50_ (μM)
**2**	32.0 ± 1.4
**23**	40.9 ± 0.3
**27**	23.1 ± 0.4
**28**	38.1 ± 2.0
**33**	17.6 ± 0.6
**34**	19.9 ± 0.7
DEX ^a^	9.6 ± 0.3

^a^ Dexamethasone, as a positive control. Values are expressed as mean ± SD (*n* = 3).

**Table 3:** The glide scores of bioactive compounds with iNOS.

Compound	Glide Score
**27**	−5.616
**33**	−5.228

## Data Availability

Not applicable.

## Supplementary Materials

The following supporting information can be downloaded at: https://www.mdpi.com/article/10.3390/molecules27061971/s1. HR-ESI-MS, UV, IR, 1D and 2D NMR spectra of **1**−**5**. Chiral HPLC separation of racemic **1**. LC/MS chromatograms of detection of **1** in the crude extract of *C. lenis*. HPLC-UV chromatograms of the derivatives of D-glucose, L-glucose, and the hydrolysate of **4**. Structures of **6**−**38**. ESI-MS and experimental and calculated ECD spectra of **22**. These materials are available online.

## Sample Availability

Samples of the compounds are not available from the authors.
