# Supplementary material for: Anti-Neuroinflammatory Components from Clausena lenis Drake
Source: Molecules. 2022 Mar 18;27(6):1971. doi: 10.3390/molecules27061971 (PMC8951546; doi:10.3390/molecules27061971)
Supplement: Supplementary file 1 [file molecules-27-01971-s001.zip › molecules-1618561-supplementary.pdf]

SUPPLEMENTARY MATERIAL

Anti-neuroinflammatory components from *Clausena lenis* Drake

Si-Si Zhu, Yi-Fan Zhang, Meng Ding, Ke-Wu Zeng, Peng-Fei Tu, Yong Jiang*

State Key Laboratory of Natural and Biomimetic Drugs, *School of Pharmaceutical Sciences*, *Peking University*, *Beijing 100191*, *People's Republic of China*

* Corresponding author.
E-mail address: yongjiang@bjmu.edu.cn. (Y. Jiang).

Contents

For compound 1
Figure S1. HR-ESI-MS of **1**.
Figure S2. UV spectrum of **1** in MeOH.
Figure S3. IR spectrum of **1**.
Figure S4. ^{1}H NMR (500 MHz) spectrum of **1** in $CDCl_3$.
Figure S5. ^{13}C NMR (125 MHz) spectrum of **1** in $CDCl_3$.
Figure S6. ^{1}H-^{1}H COSY (500 MHz) spectrum of **1** in $CDCl_3$.
Figure S7. HSQC (500 MHz) spectrum of **1** in $CDCl_3$.
Figure S8. HMBC (500 MHz) spectrum of **1** in $CDCl_3$.
Figure S9. ^{1}H NMR (500 MHz) spectrum of **1** in C_5D_5N.
Figure S10. ^{13}C NMR (125 MHz) spectrum of **1** in C_5D_5N.
Figure S11. ^{1}H-^{1}H COSY (500 MHz) spectrum of **1** in C_5D_5N.
Figure S12. HSQC (500 MHz) spectrum of **1** in C_5D_5N.
Figure S13. HMBC (500 MHz) spectrum of **1** in C_5D_5N.
Figure S14. Chiral HPLC separation of racemate **1**.
Figure S15. LC/MS chromatograms of detection of **1** in the crude extract of *C. lenis*.

For compound 2
Figure S16. HR-ESI-MS of **2**.
Figure S17. UV spectrum of **2** in MeOH.
Figure S18. IR spectrum of **2**.
Figure S19. ^{1}H NMR (500 MHz) spectrum of **2** in $CDCl_3$.
Figure S20. ^{13}C NMR (125 MHz) spectrum of **2** in $CDCl_3$.
Figure S21. ^{1}H-^{1}H COSY (500 MHz) spectrum of **2** in $CDCl_3$.
Figure S22. HSQC (500 MHz) spectrum of **2** in $CDCl_3$.
Figure S23. HMBC (500 MHz) spectrum of **2** in $CDCl_3$.

For compound 3
Figure S24. HR-ESI-MS of **3**.
Figure S25. UV spectrum of **3** in MeOH.
Figure S26. IR spectrum of **3**.
Figure S27. ^{1}H NMR (500 MHz) spectrum of **3** in $CDCl_3$.
Figure S28. ^{13}C NMR (125 MHz) spectrum of **3** in $CDCl_3$.
Figure S29. ^{1}H-^{1}H COSY (500 MHz) spectrum of **3** in $CDCl_3$.
Figure S30. HSQC (500 MHz) spectrum of **3** in $CDCl_3$.
Figure S31. HMBC (500 MHz) spectrum of **3** in $CDCl_3$.

For compound 4
Figure S32. HR-ESI-MS of **4**.
Figure S33. UV spectrum of **4** in MeOH.
Figure S34. IR spectrum of **4**.
Figure S35. ^{1}H NMR (500 MHz) spectrum of **4** in MeOD.

Figure S36. ^{13}C NMR (125 MHz) spectrum of **4** in MeOD.
Figure S37. ^{1}H-^{1}H COSY (500 MHz) spectrum of **4** in MeOD.
Figure S38. HSQC (500 MHz) spectrum of **4** in MeOD.
Figure S39. HMBC (500 MHz) spectrum of **4** in MeOD.
Figure S40. HPLC-UV (210 nm) chromatograms of the derivatives of D-glucose, L-glucose, and the hydrolysate of claulenoside A (**4**).

For compound 5
Figure S41. HR-ESI-MS of **5**.
Figure S42. UV spectrum of **5** in MeOH.
Figure S43. IR spectrum of **5**.
Figure S44. ^{1}H NMR (500 MHz) spectrum of **5** in $CDCl_3$.
Figure S45. ^{13}C NMR (125 MHz) spectrum of **5** in $CDCl_3$.
Figure S46. ^{1}H-^{1}H COSY (500 MHz) spectrum of **5** in $CDCl_3$.
Figure S47. HSQC (500 MHz) spectrum of **5** in $CDCl_3$.
Figure S48. HMBC (500 MHz) spectrum of **5** in $CDCl_3$.

For compounds 6−38
Figure S49. Chemical structures of compounds **6−38**.
Figure S50. ESI-MS of **22**.
Figure S51. Experimental and calculated ECD spectra of **22**.

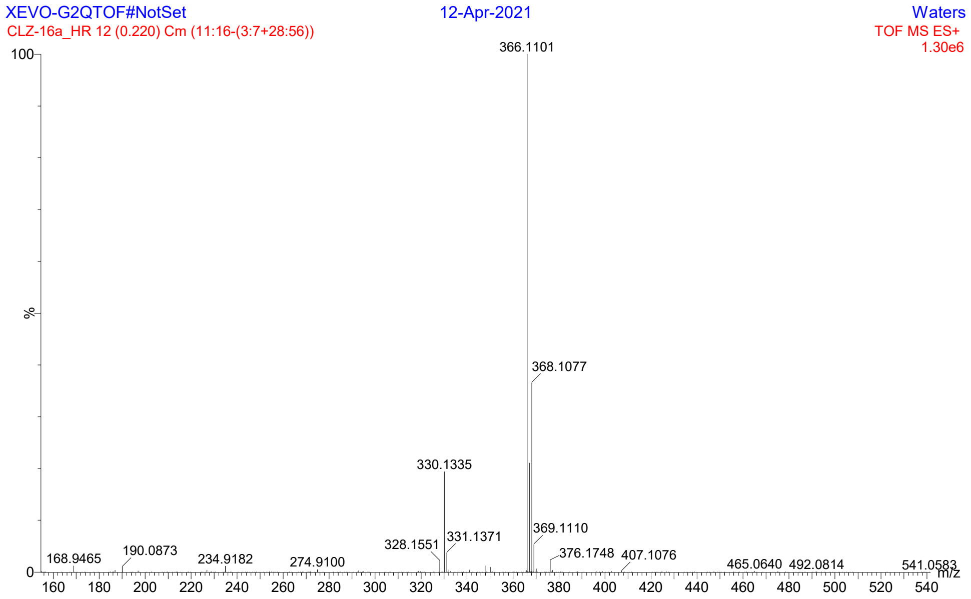

Mass	Calc. Mass	mDa	PPM	DBE	i-FIT	Norm	Conf(%)	Formula
366.1101	366.1108	-0.7	-1.9	8.5	227.6	0.000	99.98	$C_{18}H_{21}NO_5Cl$

Figure S1. HR-ESI-MS of **1**.

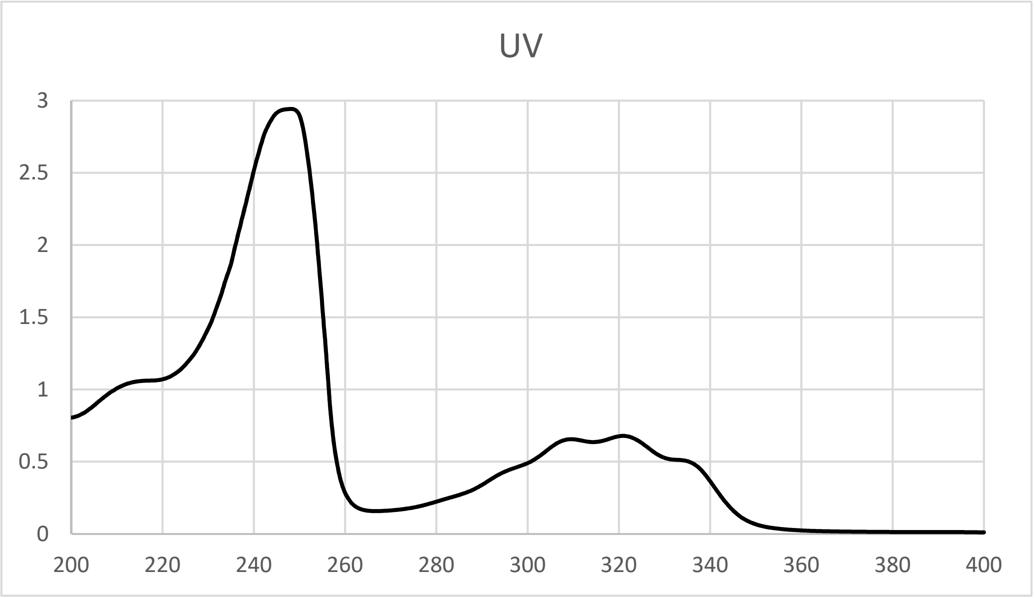

Figure S2. UV spectrum of **1** in MeOH.

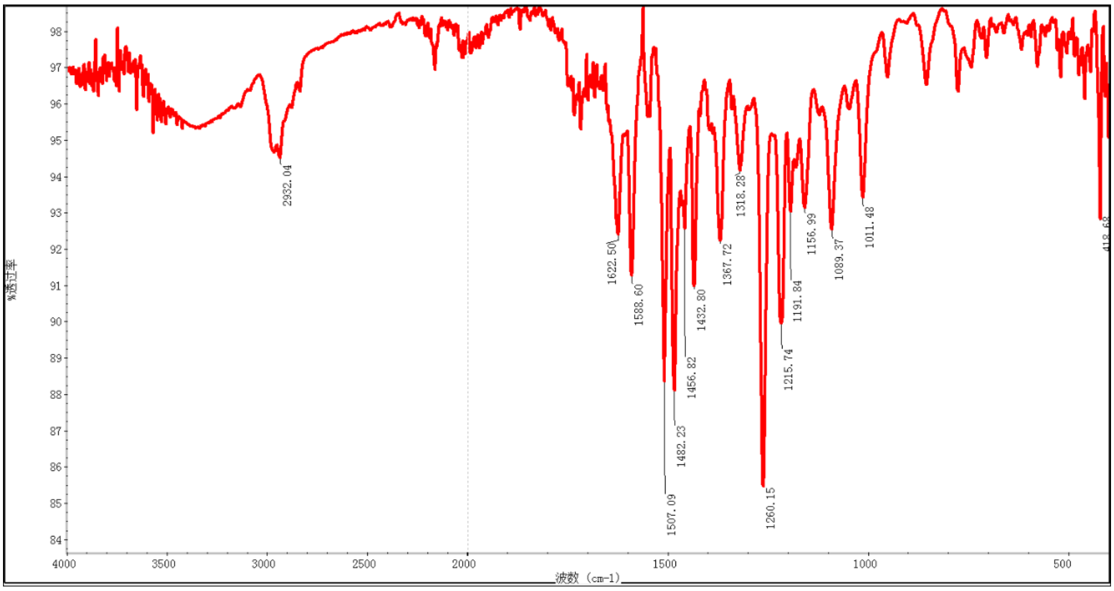

Figure S3. IR spectrum of **1**.

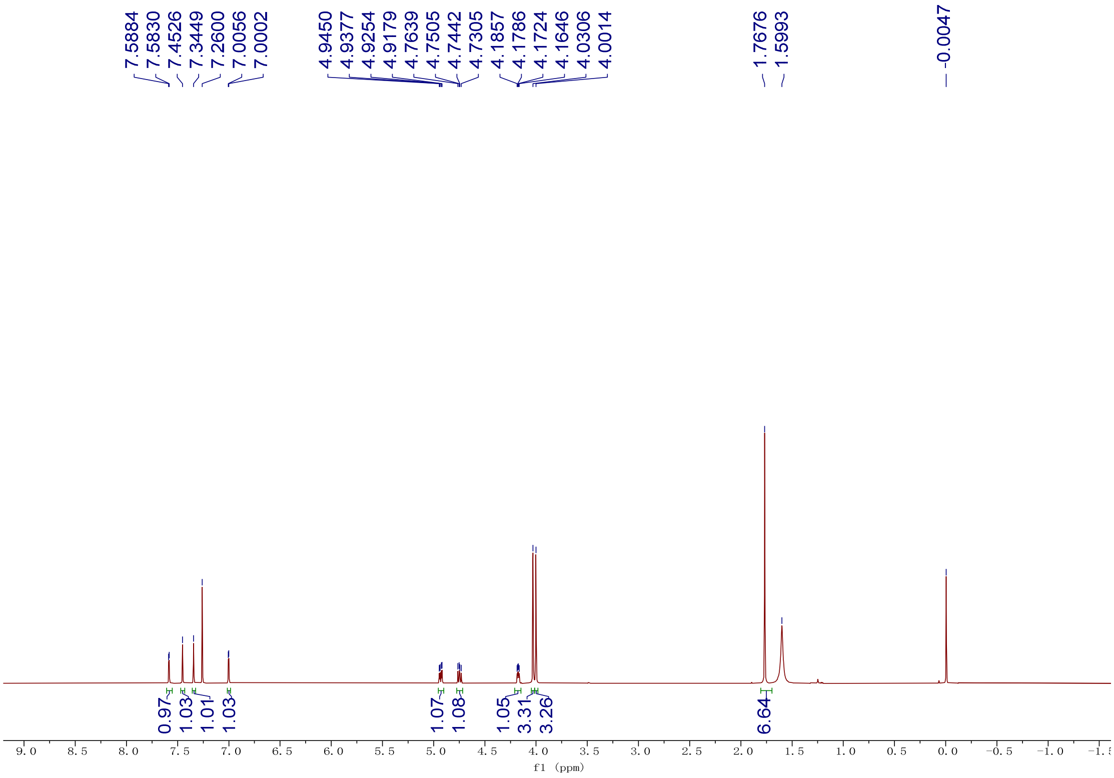

Figure S4. ^{1}H NMR (500 MHz) spectrum of **1** in $CDCl_3$.

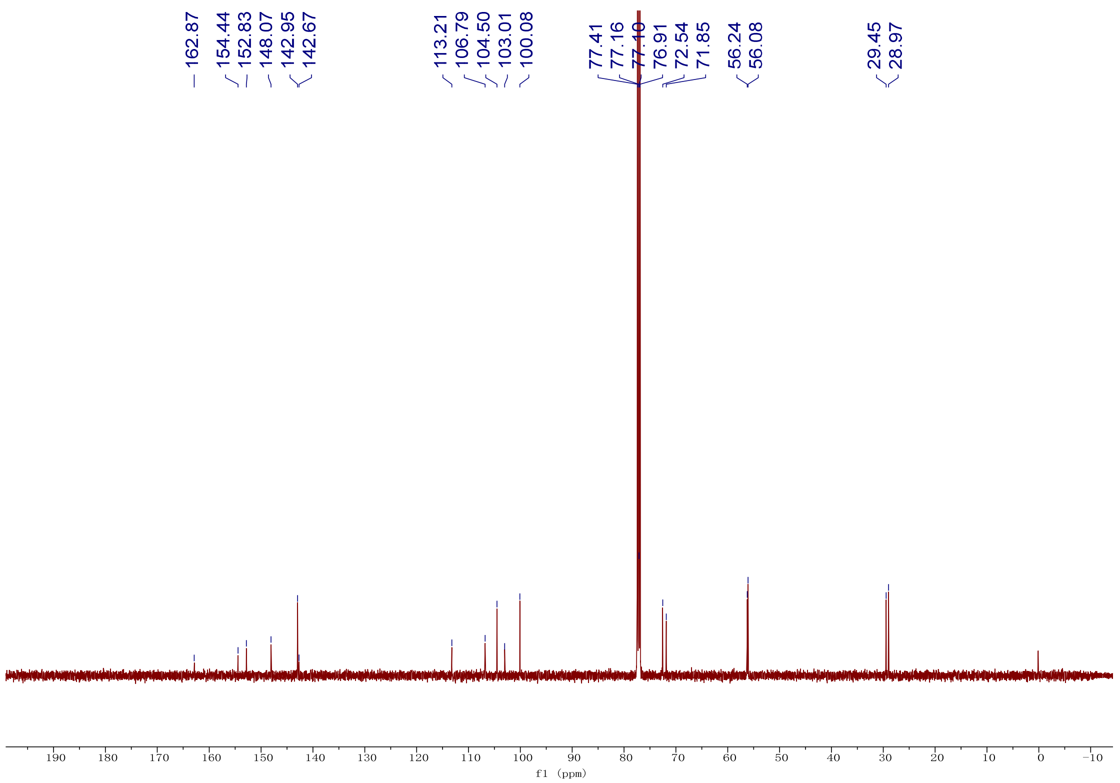

Figure S5. ^{13}C NMR (125 MHz) spectrum of **1** in $CDCl_3$.

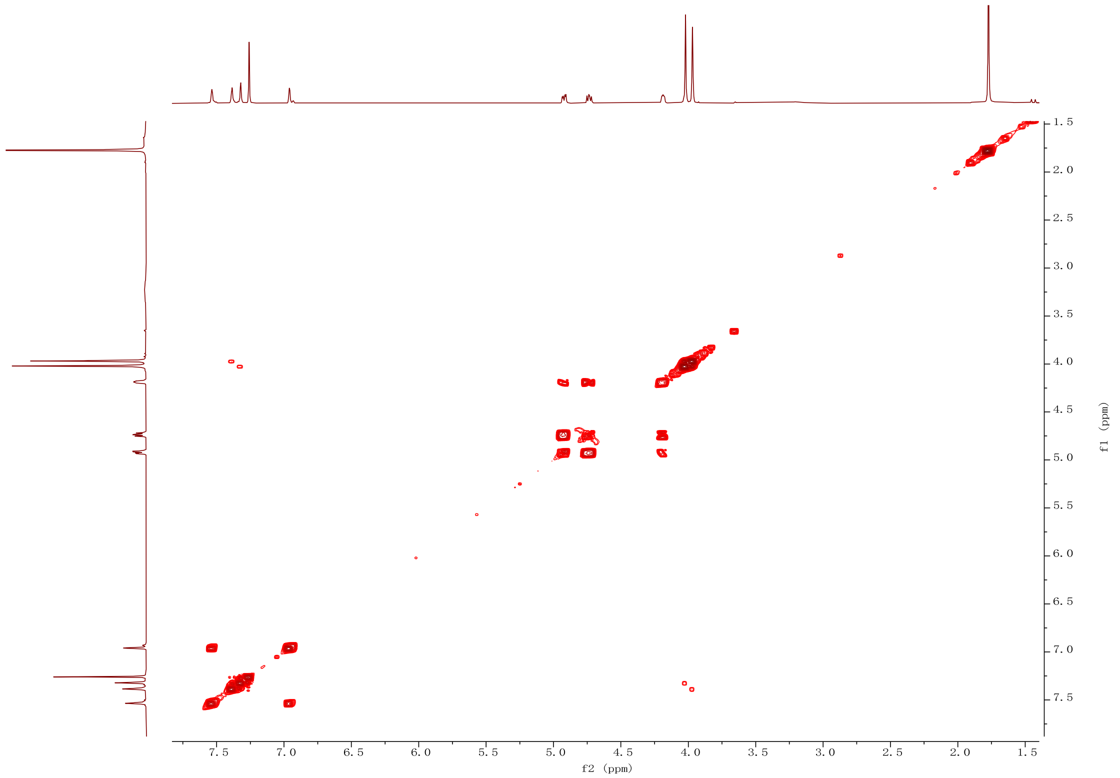

Figure S6. ^{1}H-^{1}H COSY (500 MHz) spectrum of **1** in $CDCl_3$.

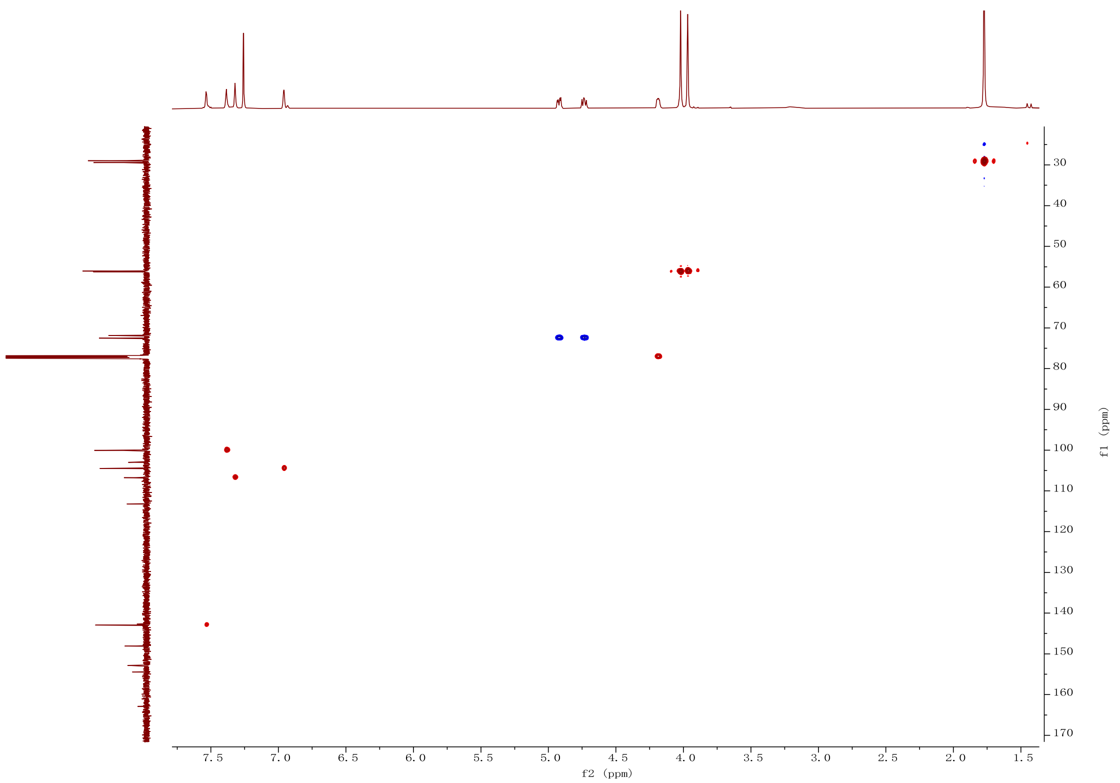

Figure S7. HSQC (500 MHz) spectrum of **1** in $CDCl_3$.

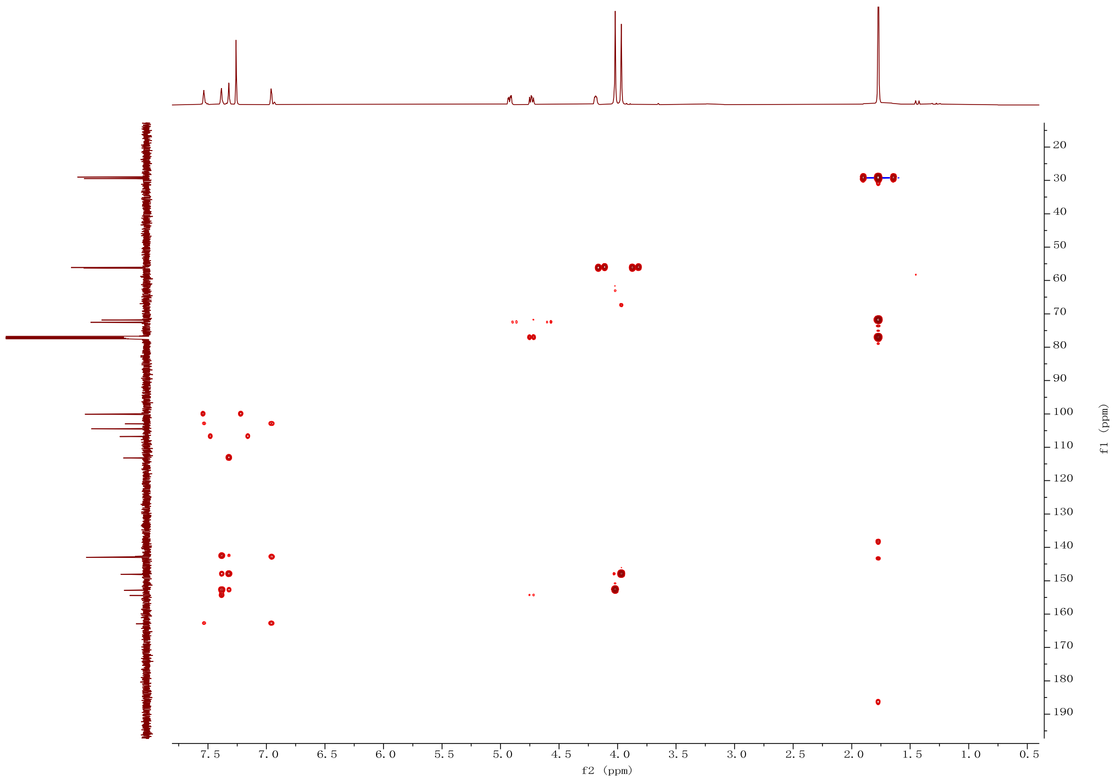

Figure S8. HMBC (500 MHz) spectrum of **1** in $CDCl_3$.

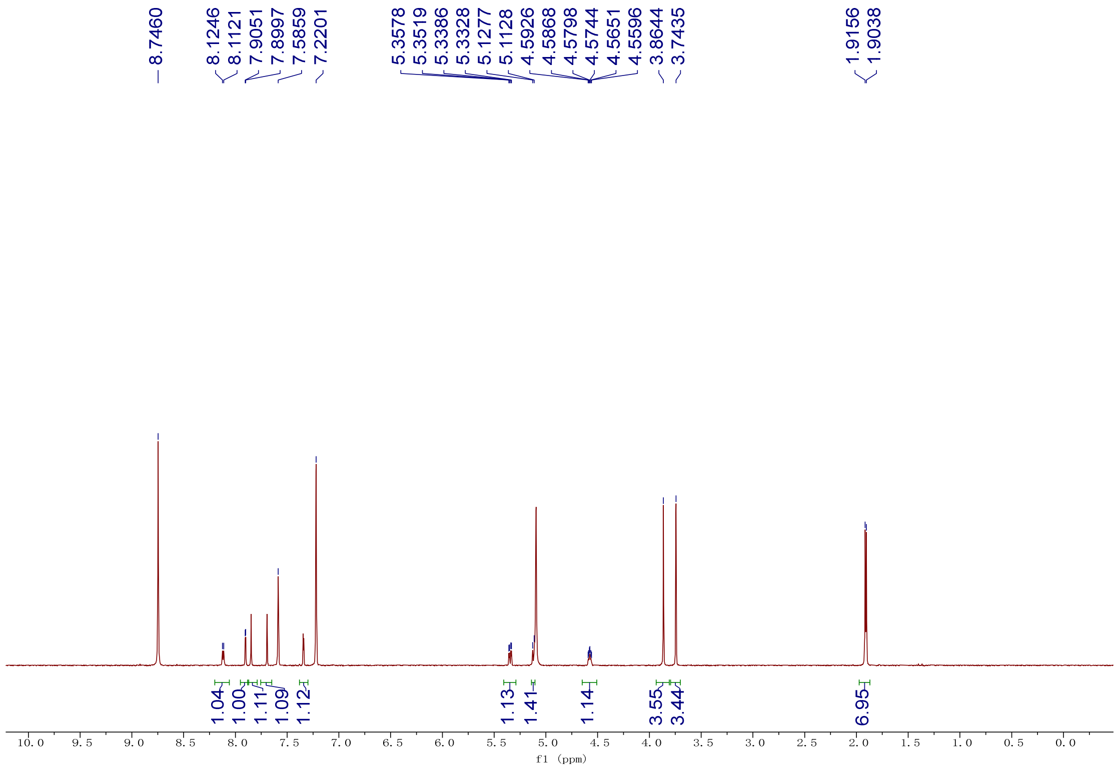

Figure S9. ^{1}H NMR (500 MHz) spectrum of **1** in C_5D_5N.

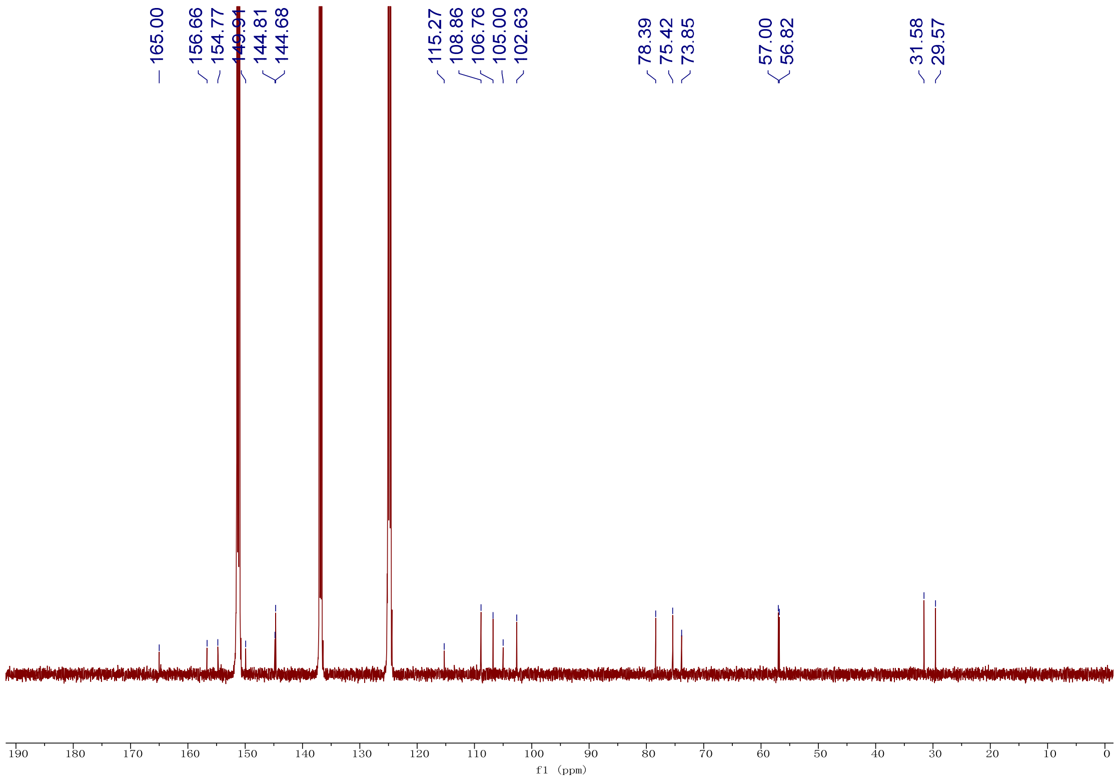

Figure S10. ^{13}C NMR (125 MHz) spectrum of **1** in C_5D_5N.

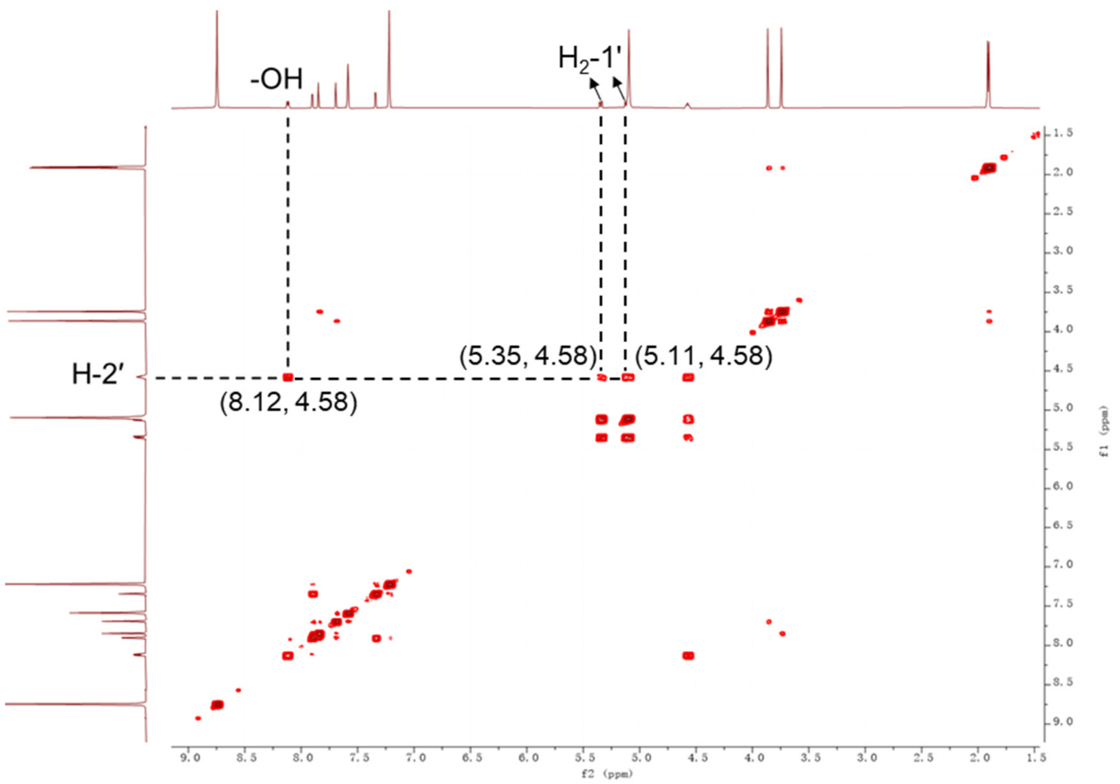

Figure S11. ^{1}H-^{1}H COSY (500 MHz) spectrum of **1** in C_5D_5N.

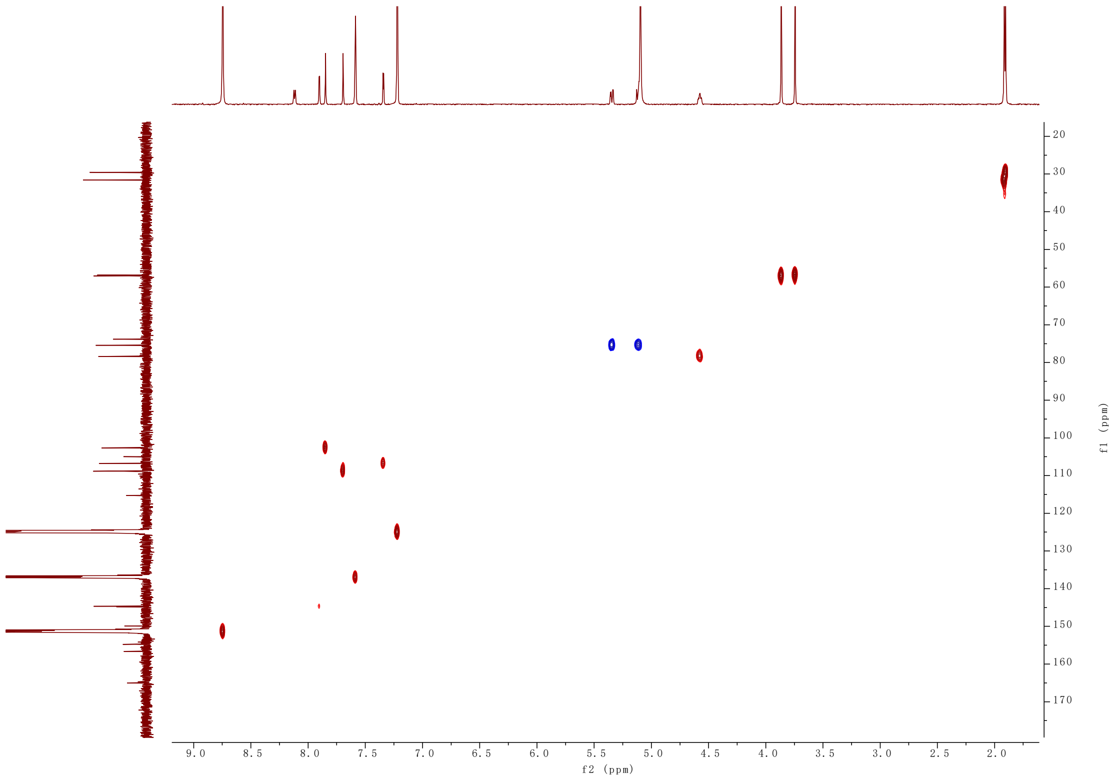

Figure S12. HSQC (500 MHz) spectrum of **1** in C_5D_5N.

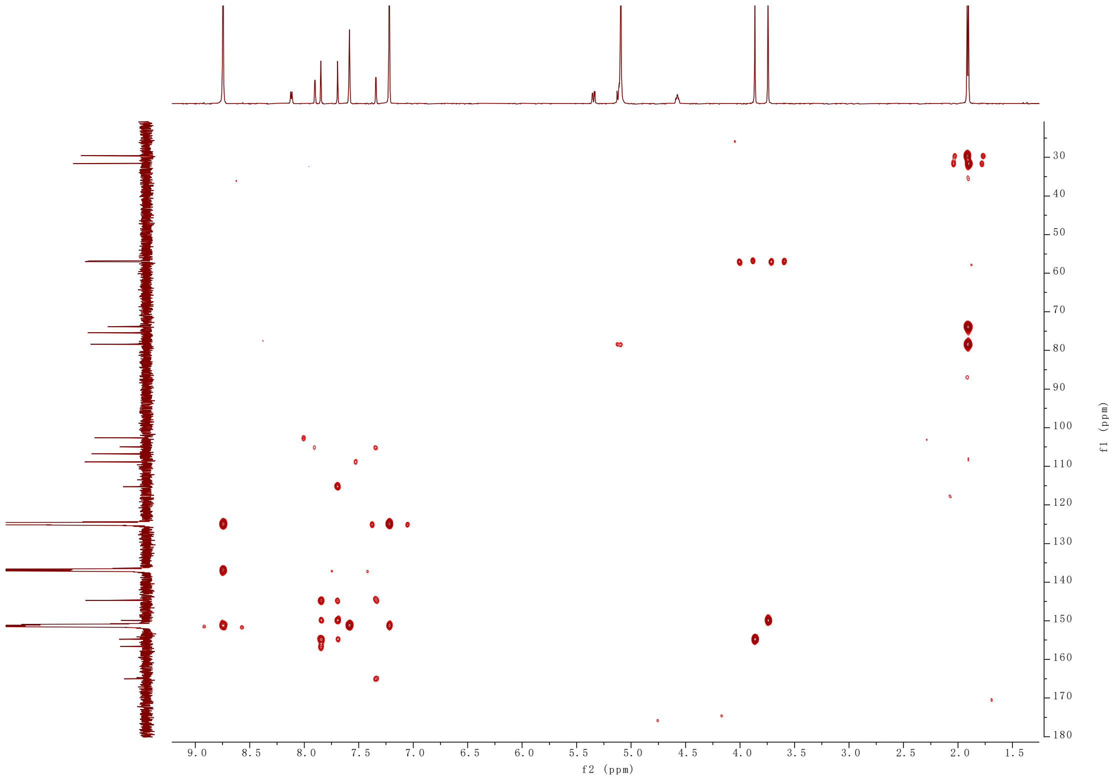

Figure S13. HMBC (500 MHz) spectrum of **1** in C_5D_5N.

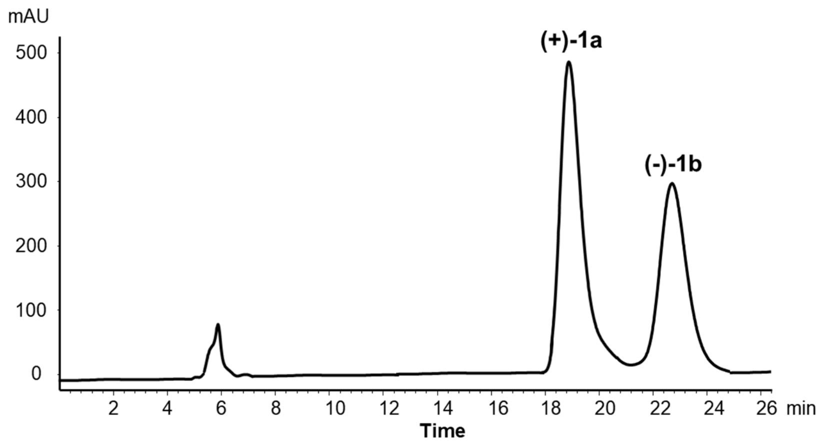

Figure S14. Chiral HPLC separation of racemate **1** (*n*-hexane/isopropanol, 85:15; T = 30 ℃; flow rate: 3.0 mL/min; wavelength: 254 nm).

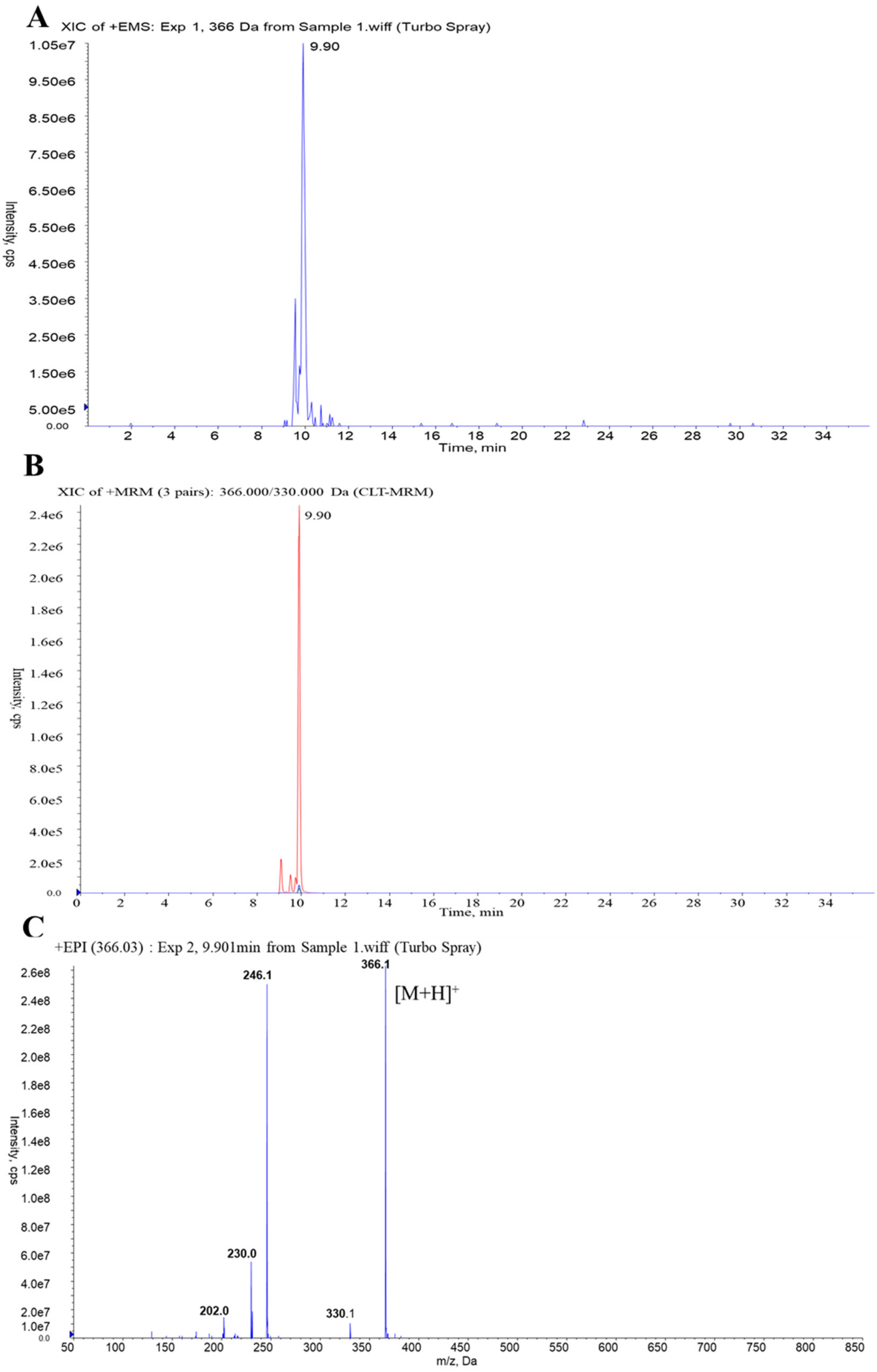

Figure S15. LC/MS chromatograms of detection of **1** in the crude extract of *C. lenis*. (A: EIC of **1** (*m*/*z* 366); B: XIC of **1** (*m*/*z* 366) from the crude extract of *C. lenis* under the MRM mode; C: The fragmentation behavior of **1** (*m*/*z* 366).

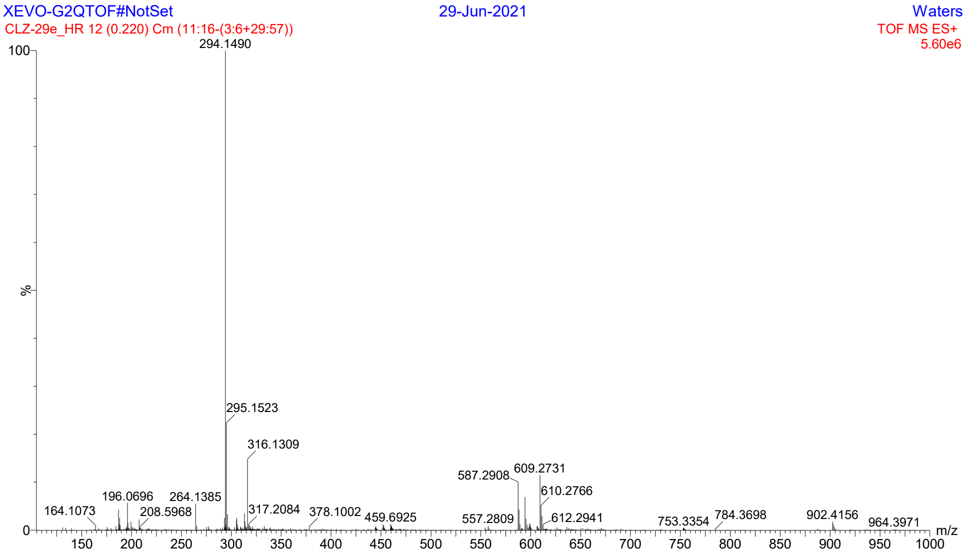

Mass	Calc. Mass	mDa	PPM	DBE	i-FIT	Norm	Conf(%)	Formula
294.1490	294.1494	-0.4	-1.4	10.5	515.3	n/a	n/a	$C_{19}H_{20}NO_2$

Figure S16. HR-ESI-MS of **2**.

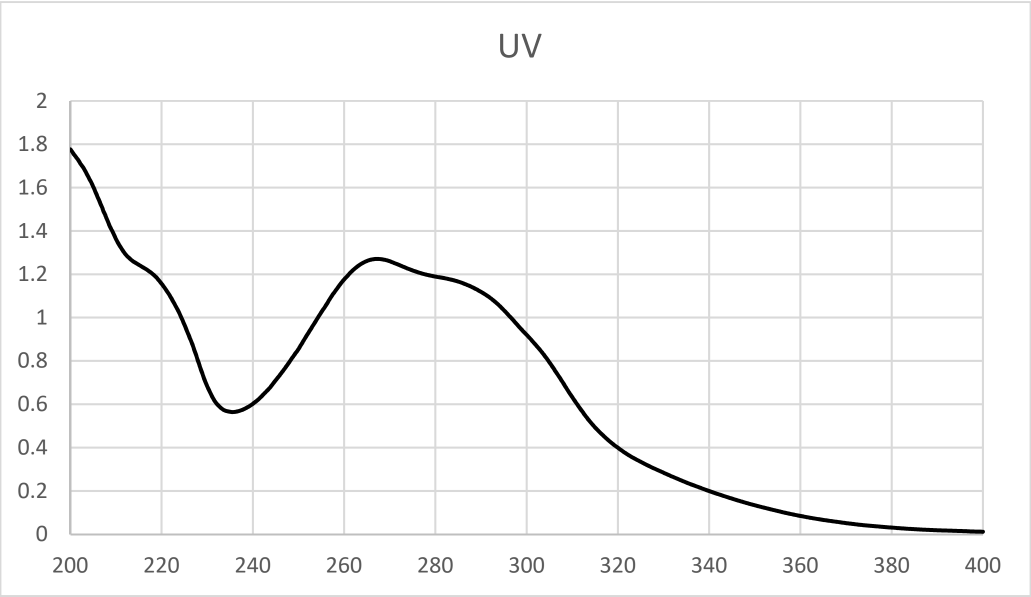

Figure S17. UV spectrum of **2** in MeOH.

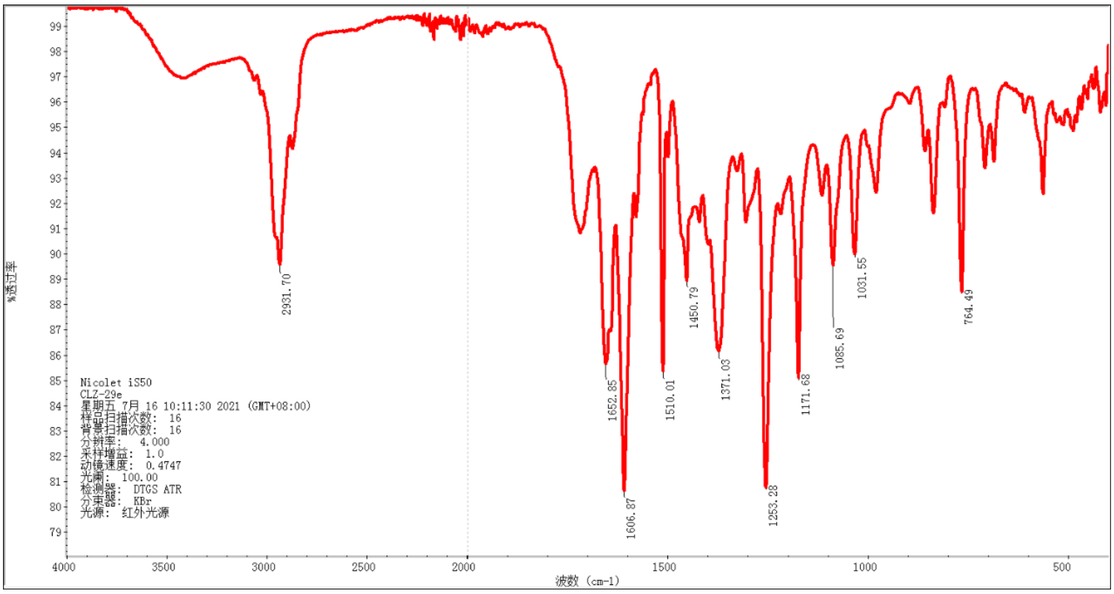

Figure S18. IR spectrum of **2**.

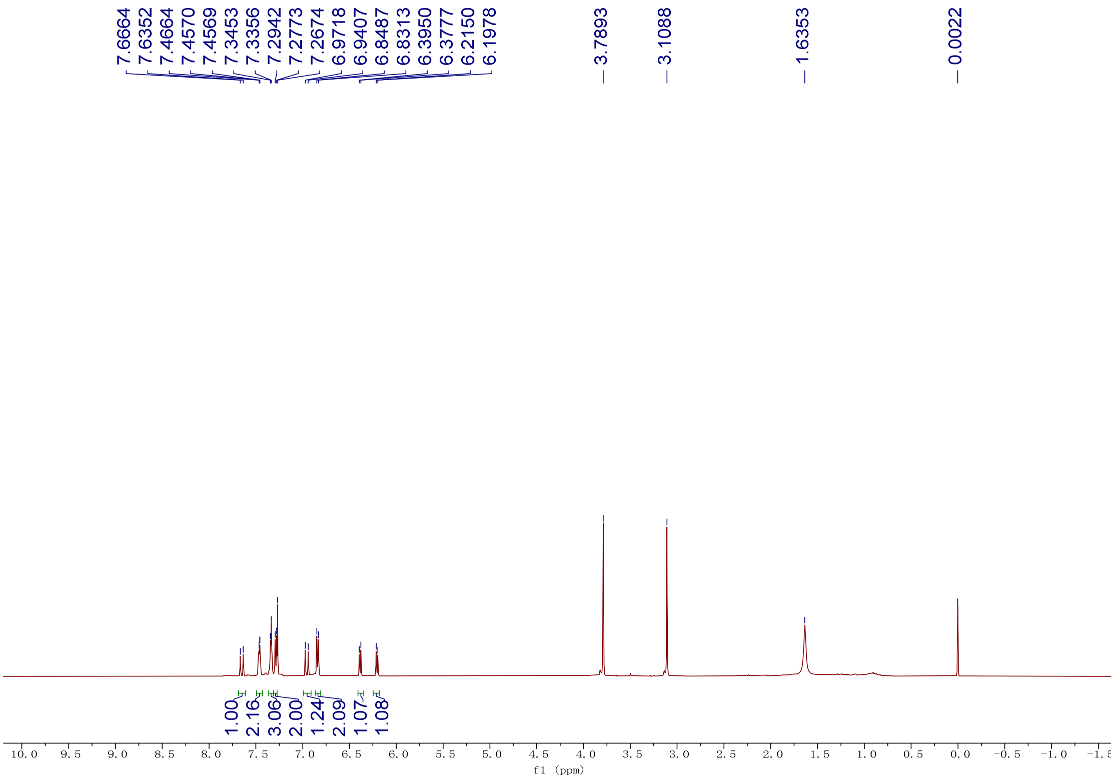

Figure S19. ^{1}H NMR (500 MHz) spectrum of **2** in $CDCl_3$.

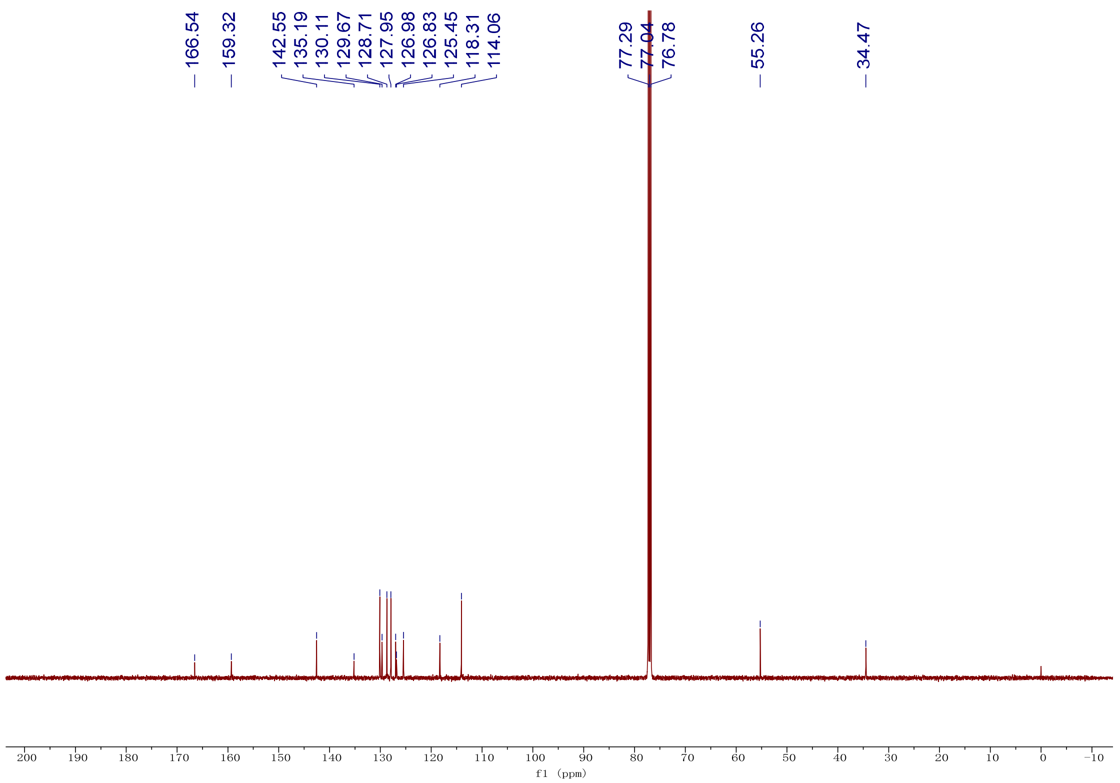

Figure S20. ^{13}C NMR (125 MHz) spectrum of **2** in $CDCl_3$.

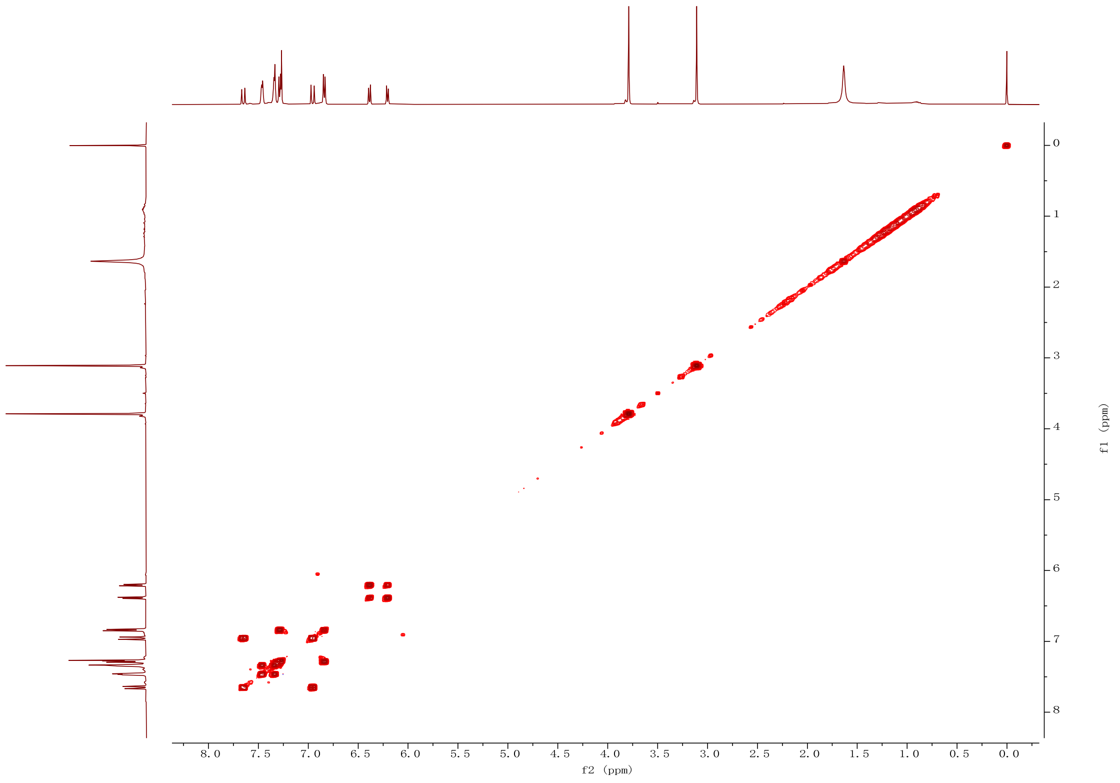

Figure S21. ^{1}H-^{1}H COSY (500 MHz) spectrum of **2** in $CDCl_3$.

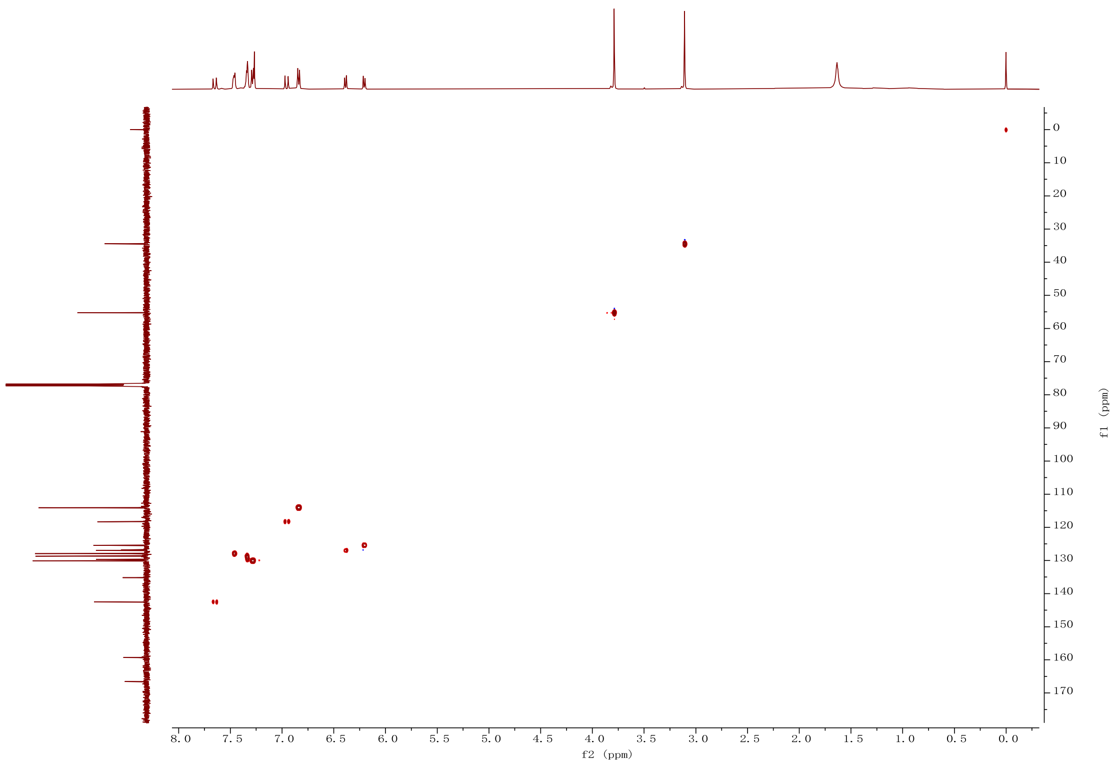

Figure S22. HSQC (500 MHz) spectrum of **2** in $CDCl_3$.

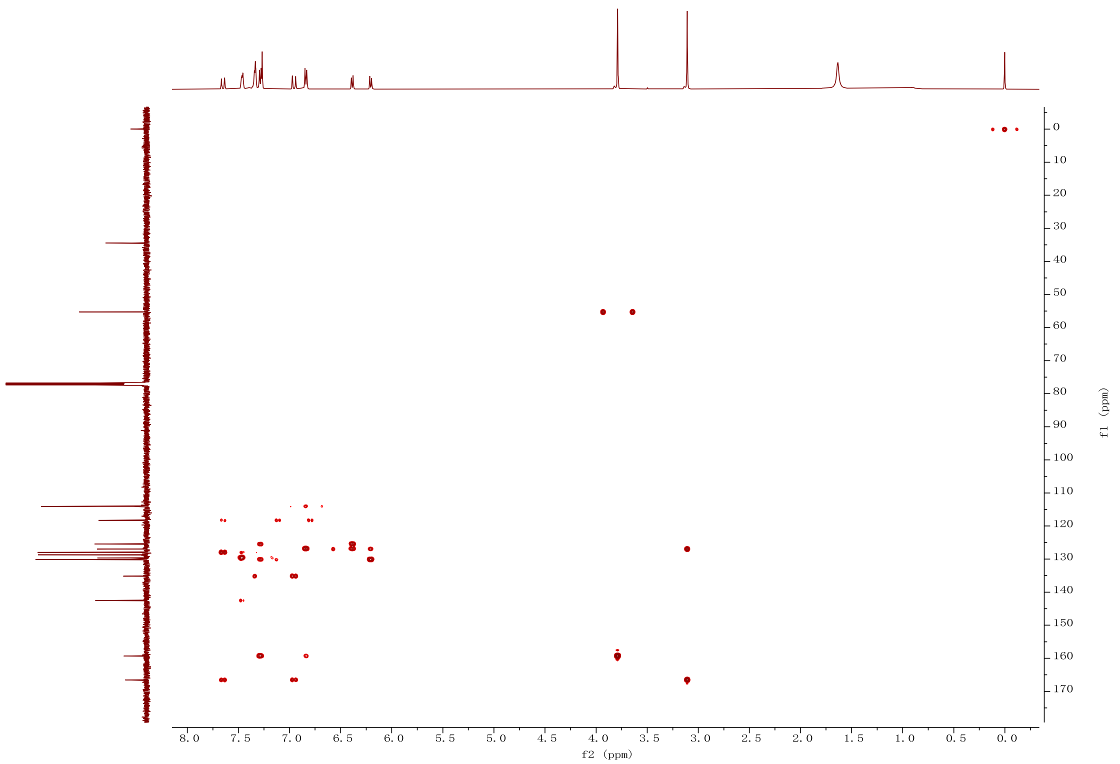

Figure S23. HMBC (500 MHz) spectrum of **2** in $CDCl_3$.

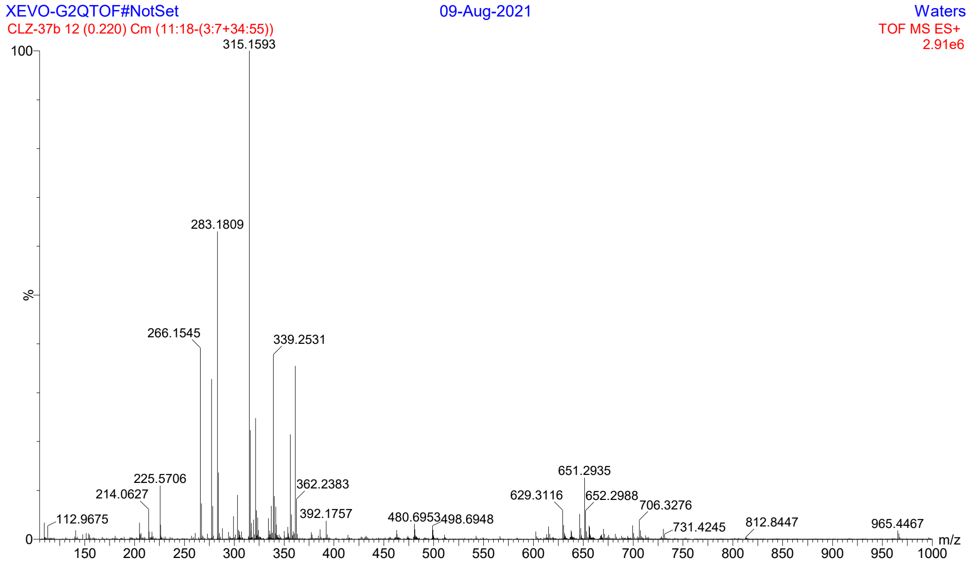

Mass	Calc. Mass	mDa	PPM	DBE	i-FIT	Norm	Conf(%)	Formula
315.1594	315.1596	-0.2	-0.6	9.5	205.4	0.017	98.32	$C_{19}H_{23}O_4$

Figure S24. HR-ESI-MS of **3**.

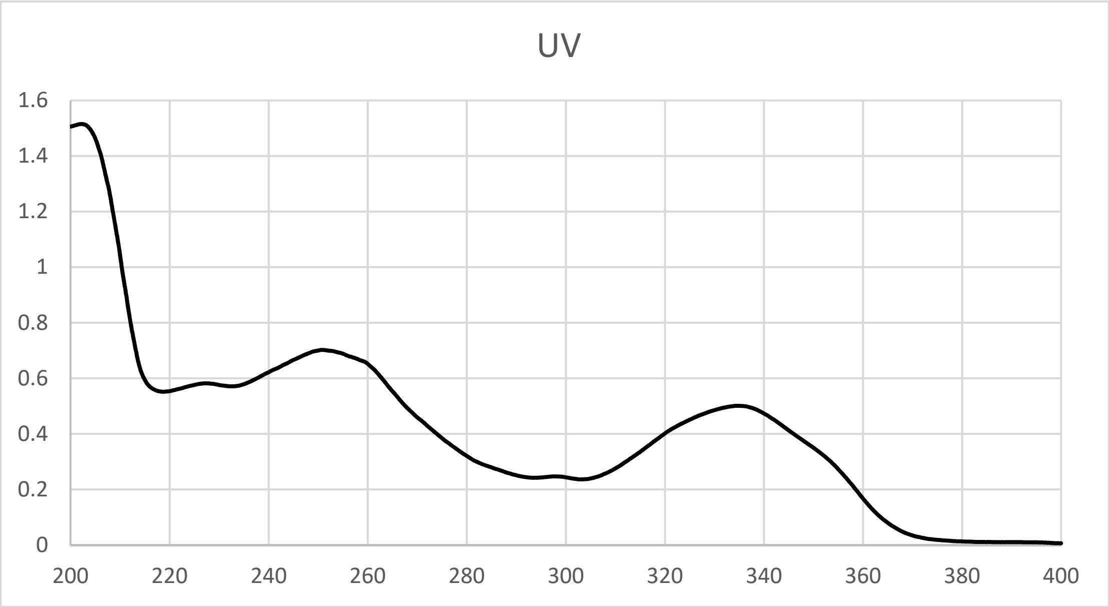

Figure S25. UV spectrum of **3** in MeOH.

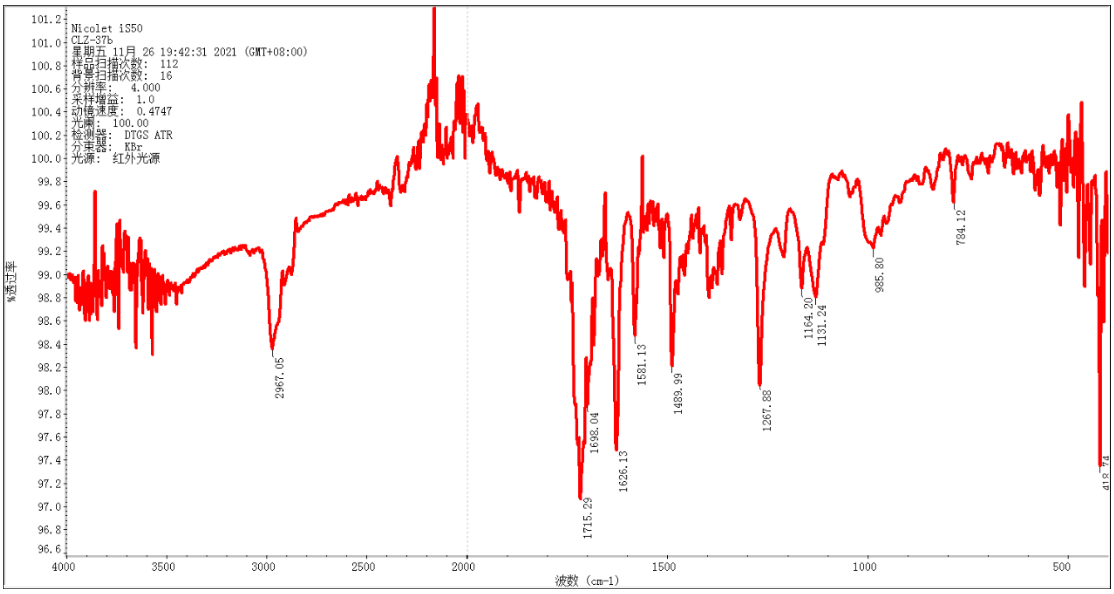

Figure S26. IR spectrum of **3**.

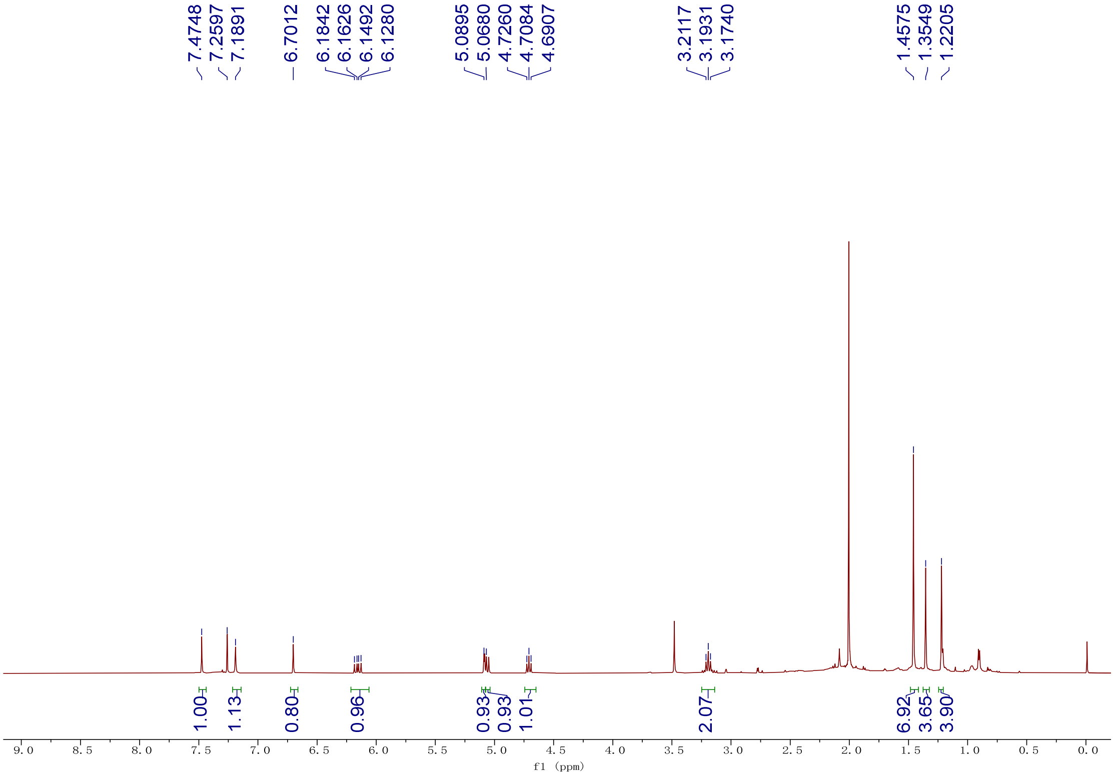

Figure S27. ^{1}H NMR (500 MHz) spectrum of **3** in $CDCl_3$.

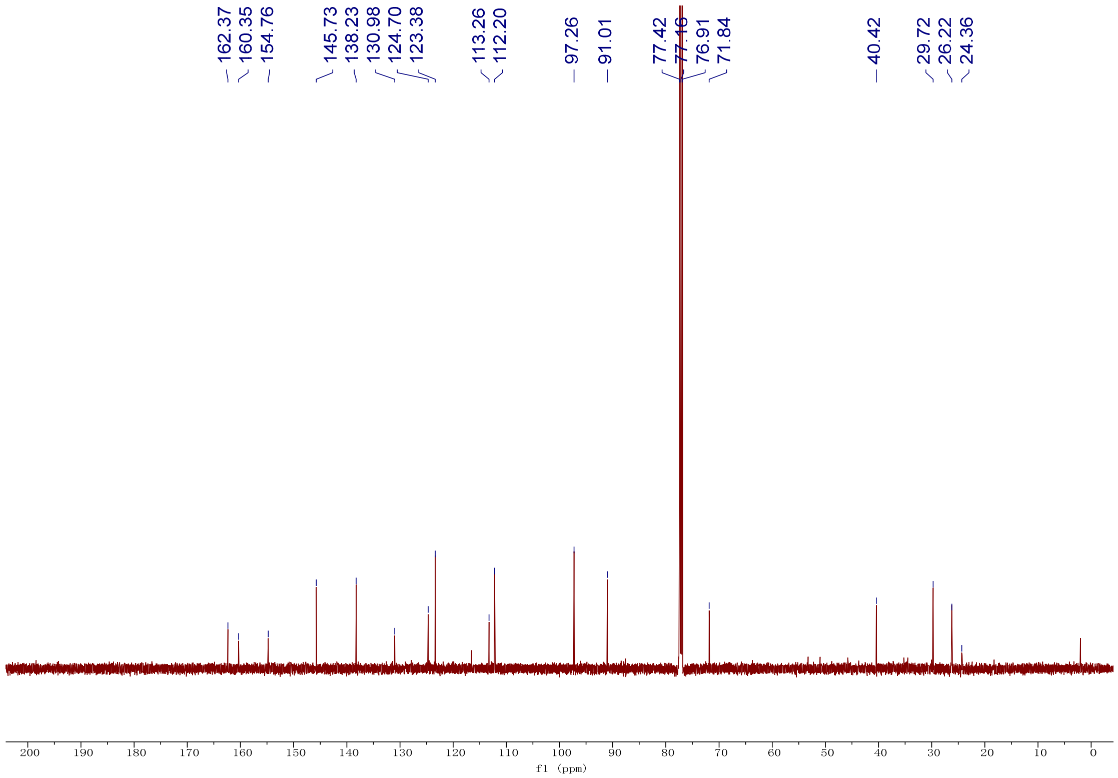

Figure S28. ^{13}C NMR (125 MHz) spectrum of **3** in $CDCl_3$.

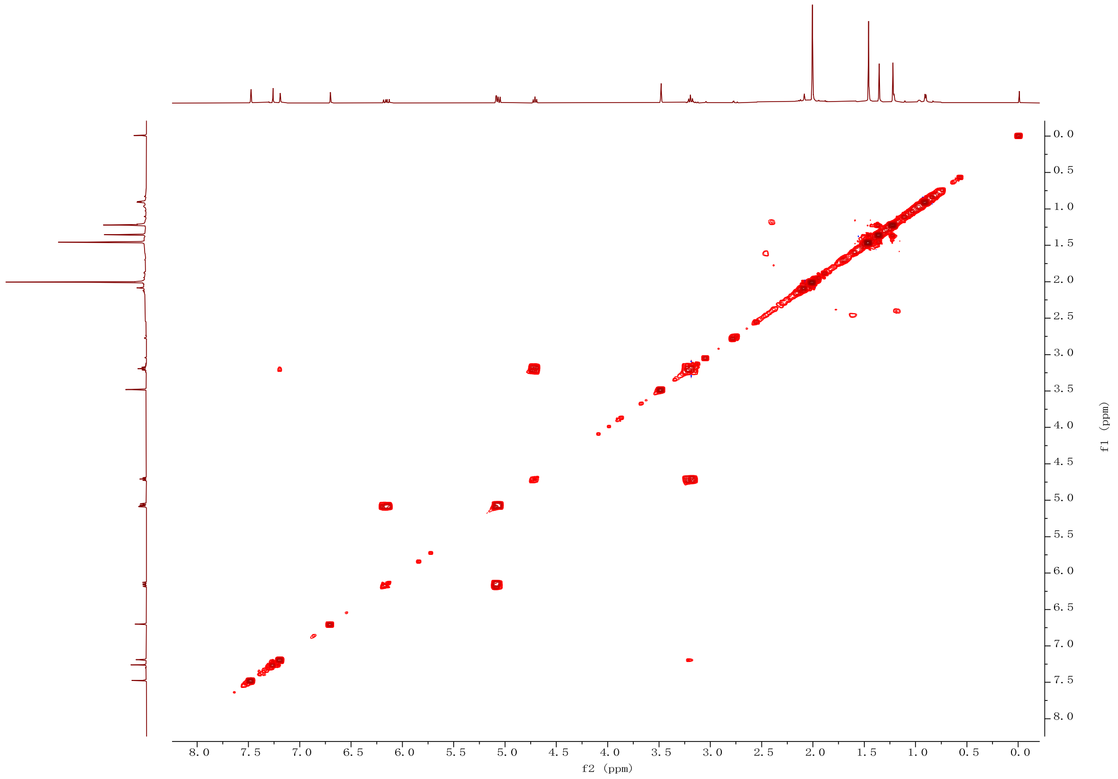

Figure S29. ^{1}H-^{1}H COSY (500 MHz) spectrum of **3** in $CDCl_3$.

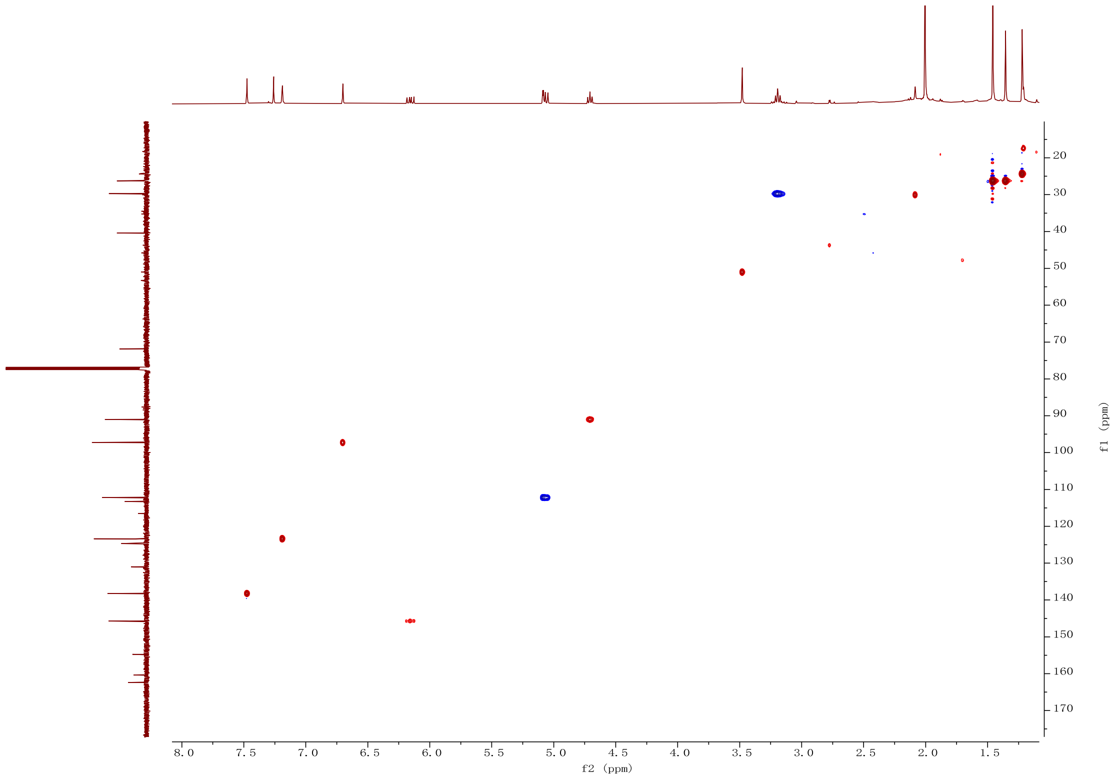

Figure S30. HSQC (500 MHz) spectrum of **3** in $CDCl_3$.

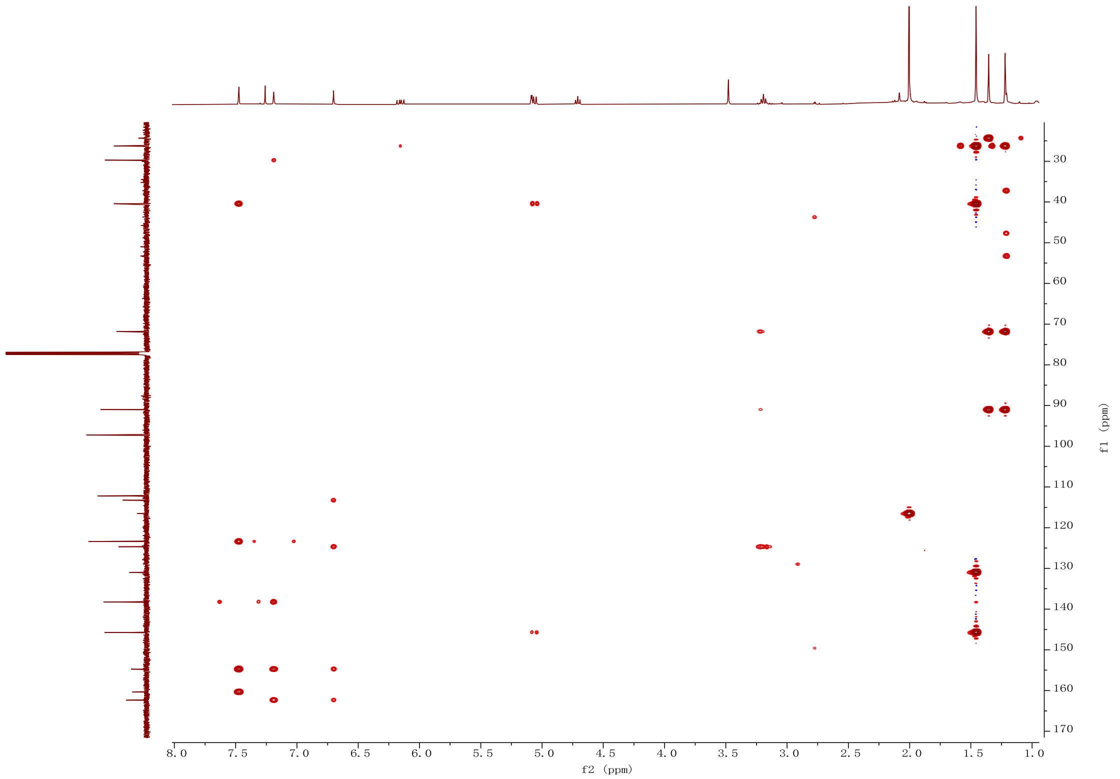

Figure S31. HMBC (500 MHz) spectrum of **3** in $CDCl_3$.

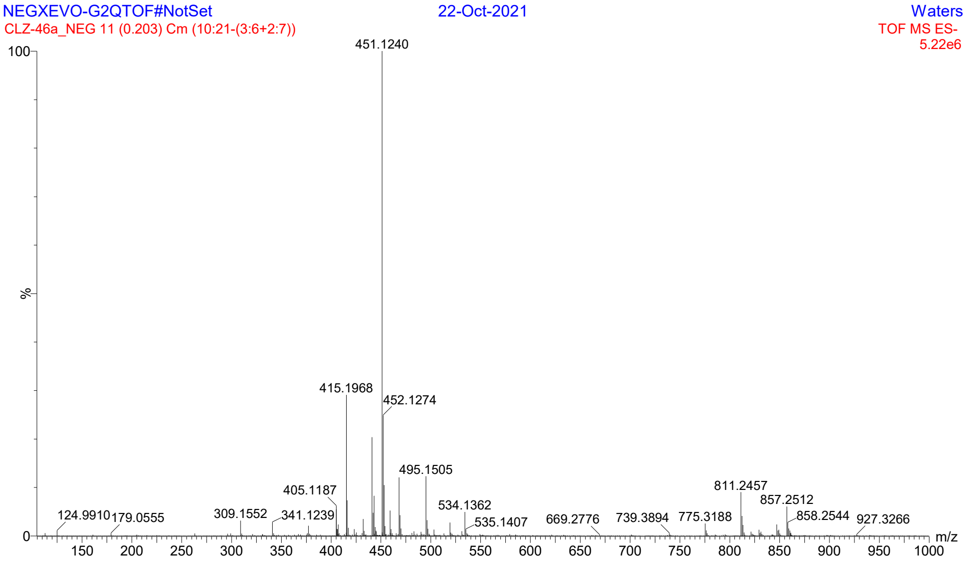

Mass	Calc. Mass	mDa	PPM	DBE	i-FIT	Norm	Conf(%)	Formula
405.1187	405.1186	0.1	0.2	10.5	491.6	n/a	n/a	$C_{20}H_{21}O_9$

Figure S32. HR-ESI-MS of **4**.

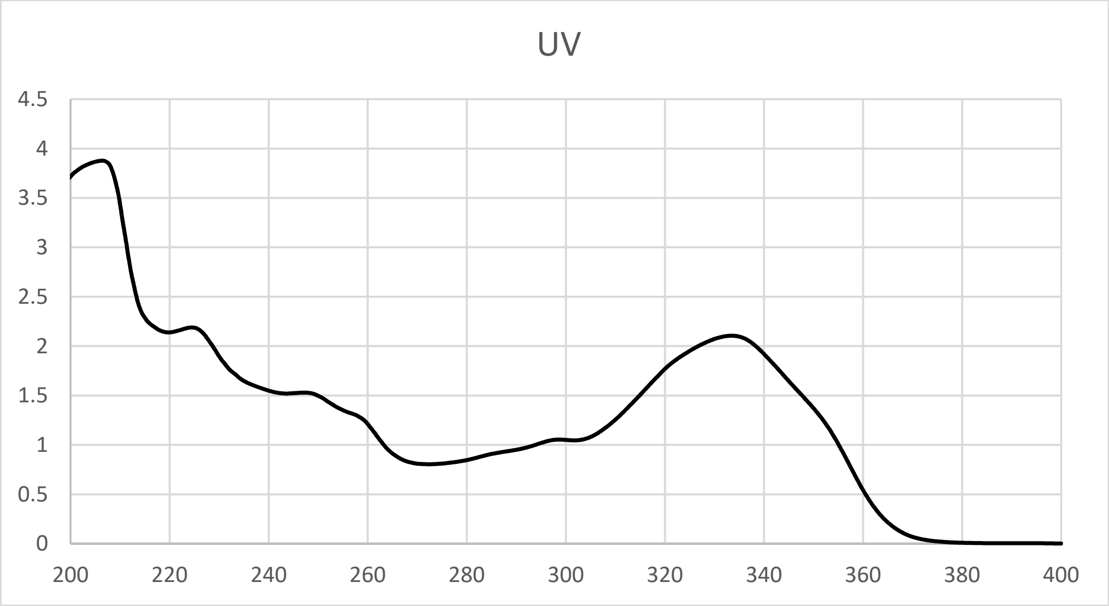

Figure S33. UV spectrum of **4** in MeOH.

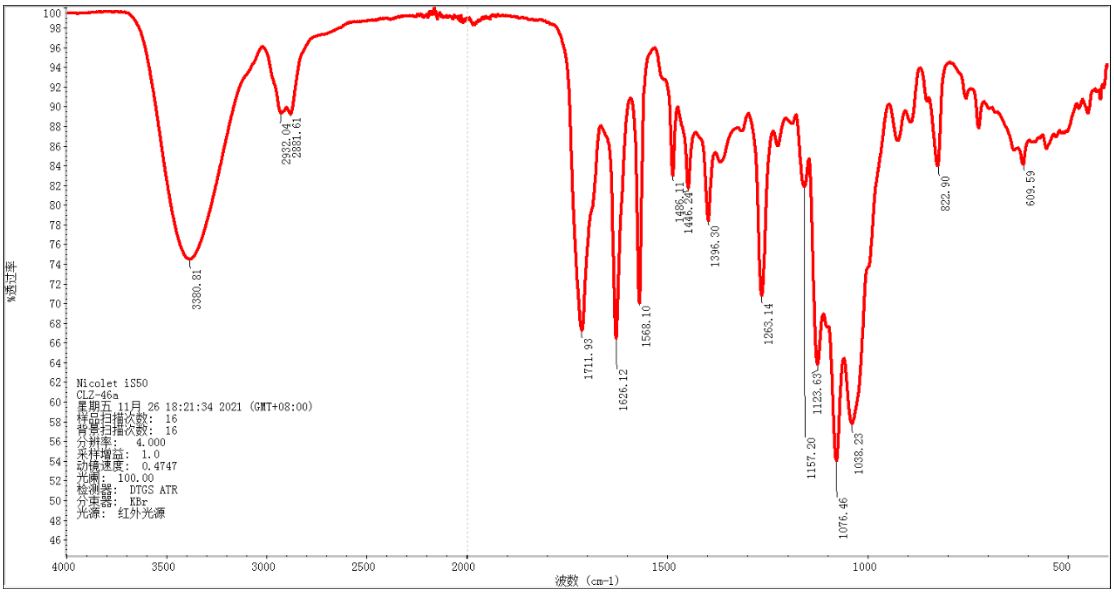

Figure S34. IR spectrum of **4**.

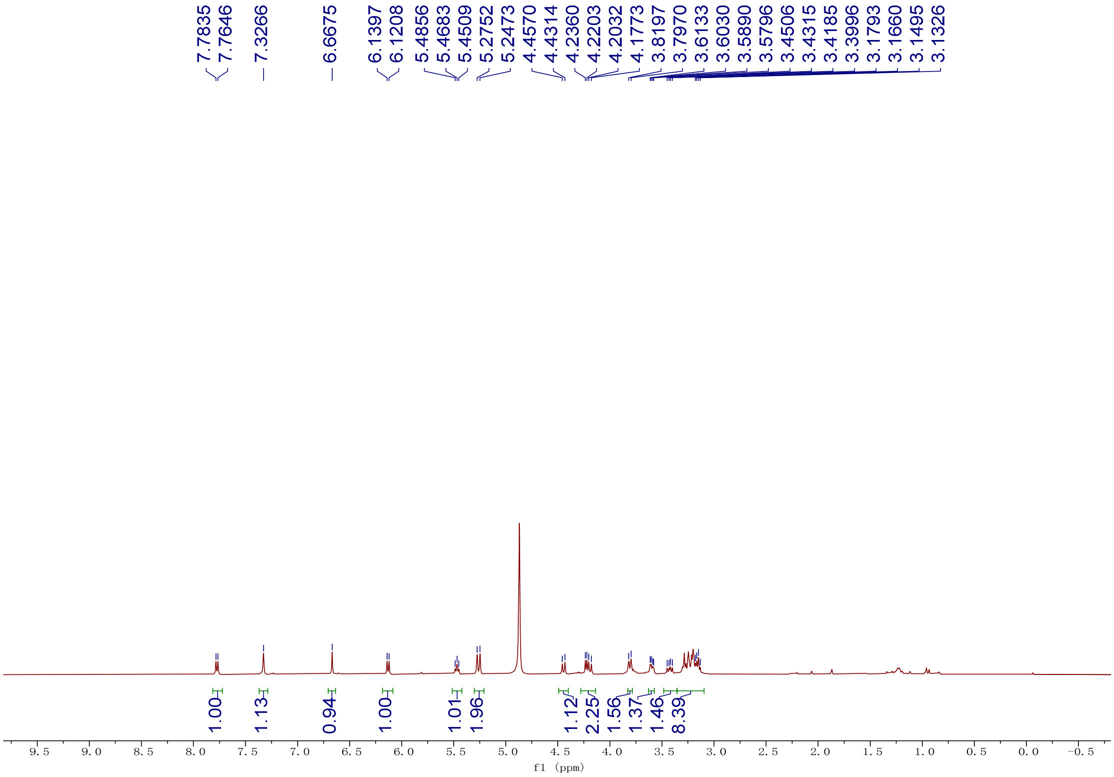

Figure S35. ^{1}H NMR (500 MHz) spectrum of **4** in MeOD.

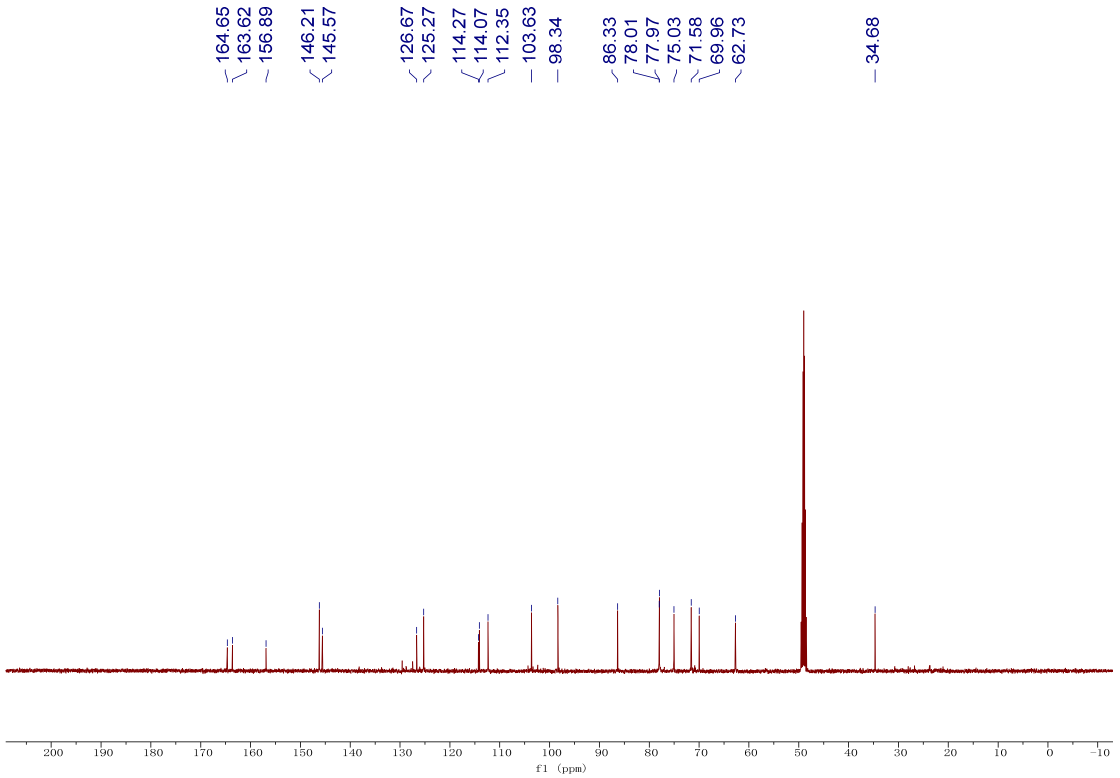

Figure S36. ^{13}C NMR (125 MHz) spectrum of **4** in MeOD.

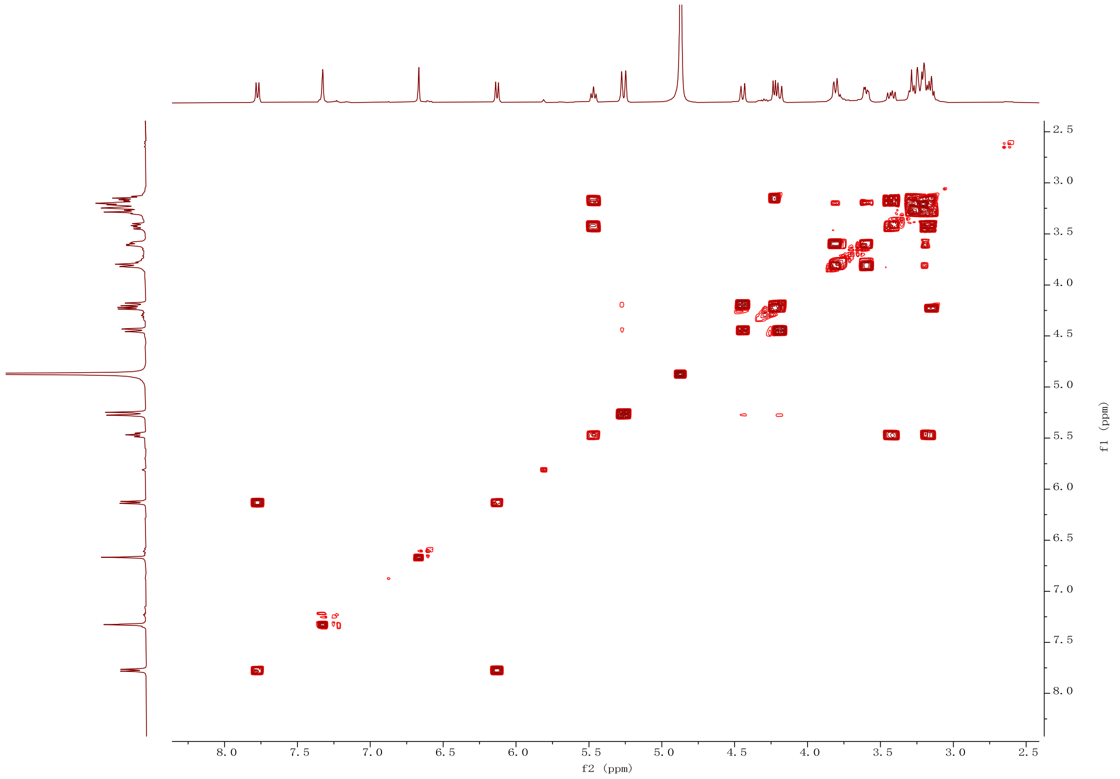

Figure S37. ^{1}H-^{1}H COSY (500 MHz) spectrum of **4** in MeOD.

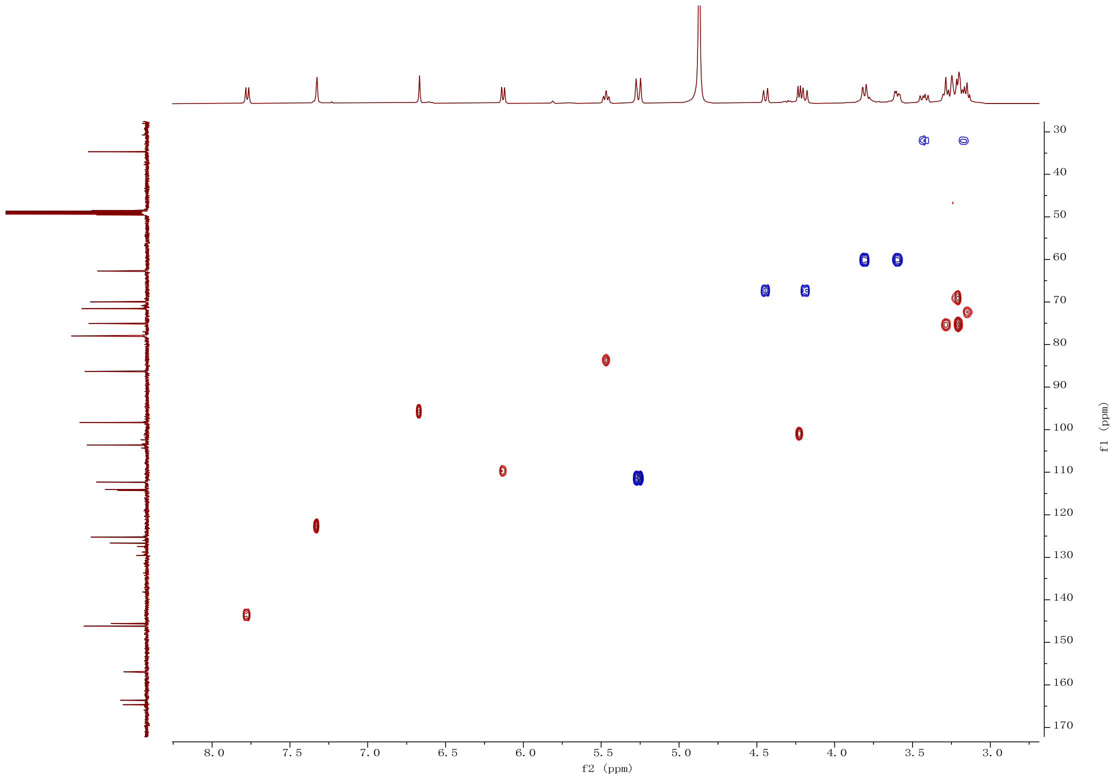

Figure S38. HSQC (500 MHz) spectrum of **4** in MeOD.

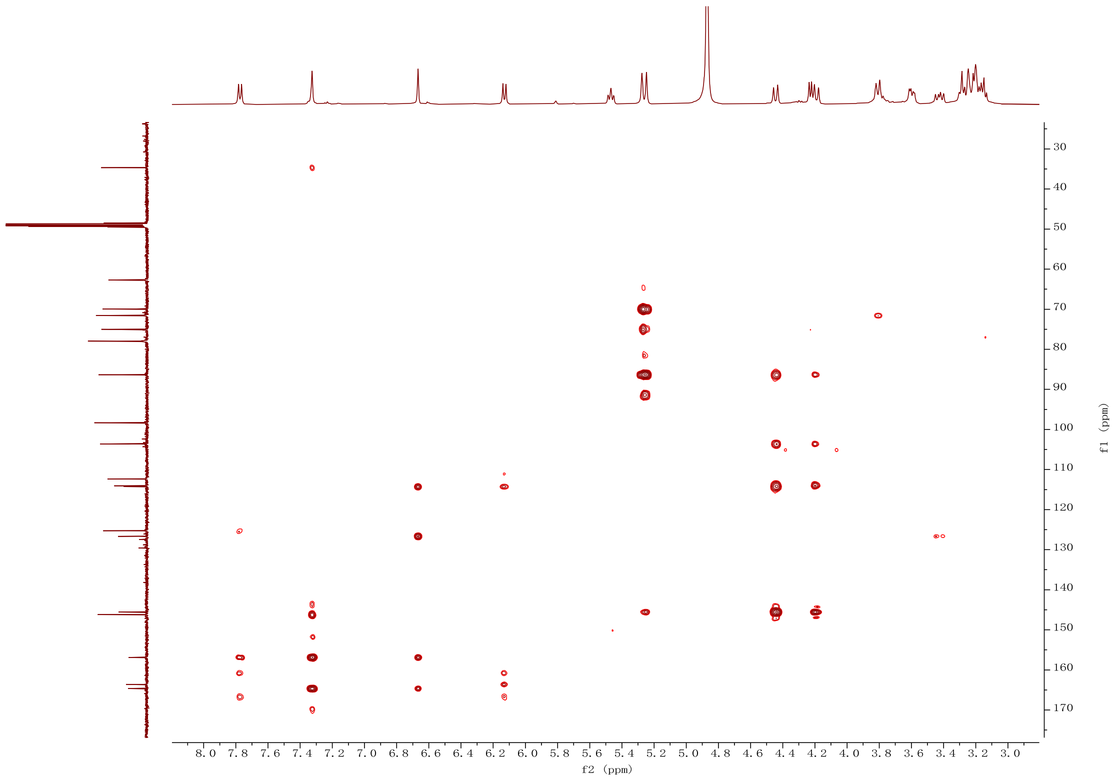

Figure S39. HMBC (500 MHz) spectrum of **4** in MeOD.

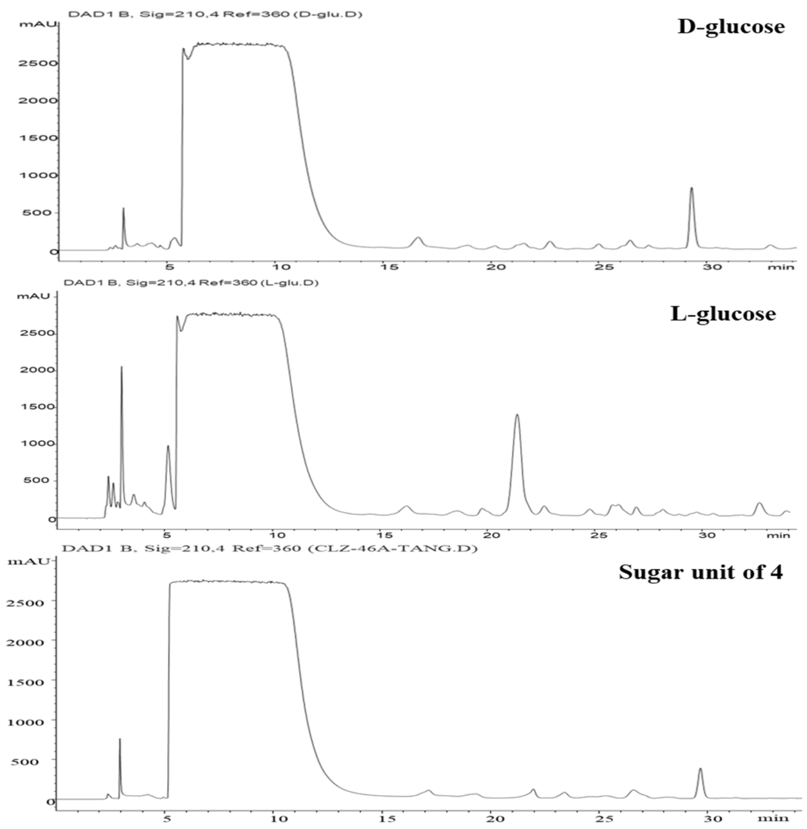

Figure S40. HPLC-UV (210 nm) chromatograms of the derivatives of D-glucose, L-glucose, and the sugar units of claulenoside A (**4**).

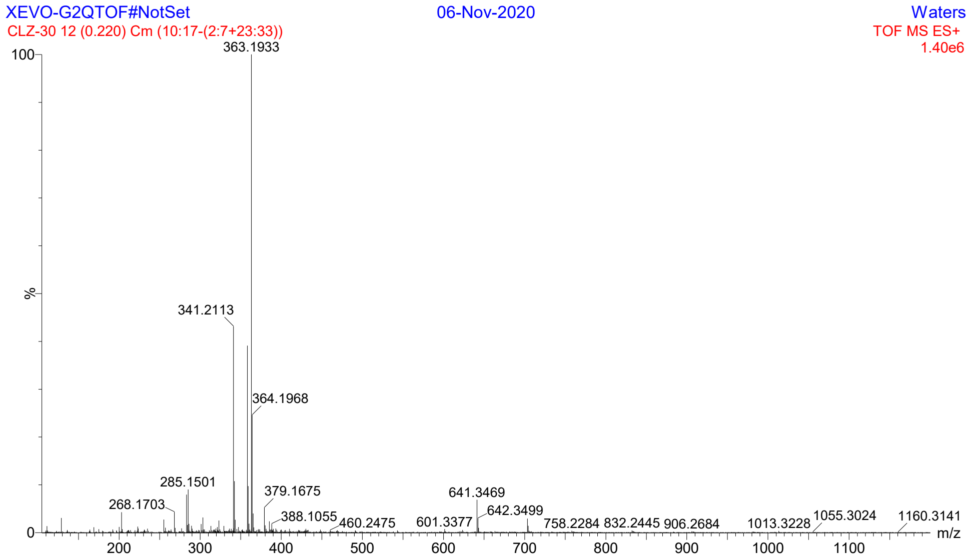

Mass	Calc. Mass	mDa	PPM	DBE	i-FIT	Norm	Conf(%)	Formula
363.1933	363.1936	-0.3	-0.8	8.5	67.4	n/a	n/a	$C_{22}H_{28}O_3Na$

Figure S41. HR-ESI-MS of **5**.

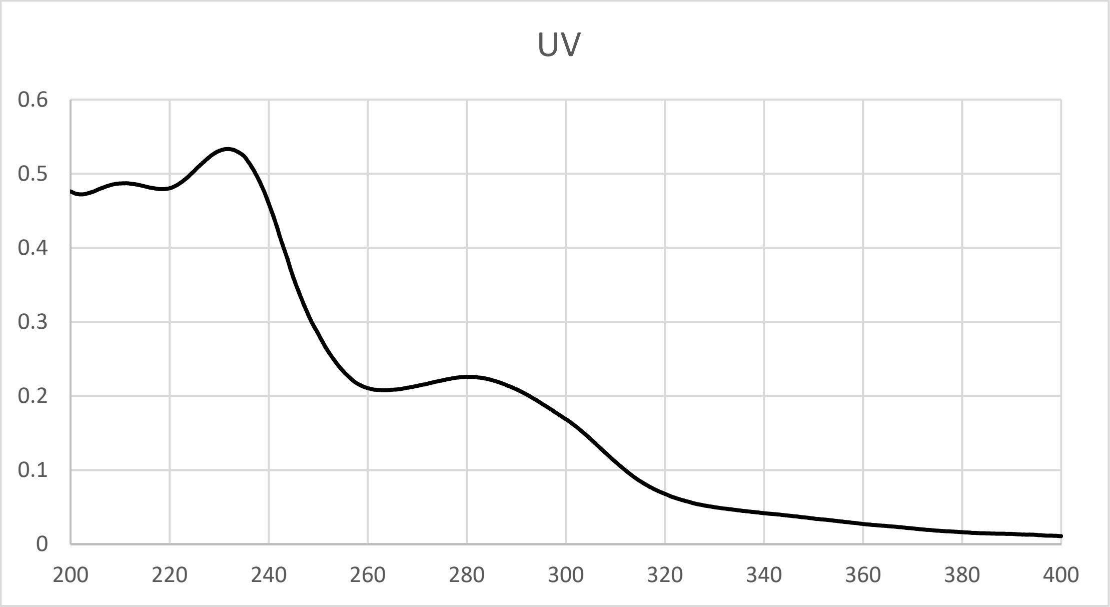

Figure S42. UV spectrum of **5** in MeOH.

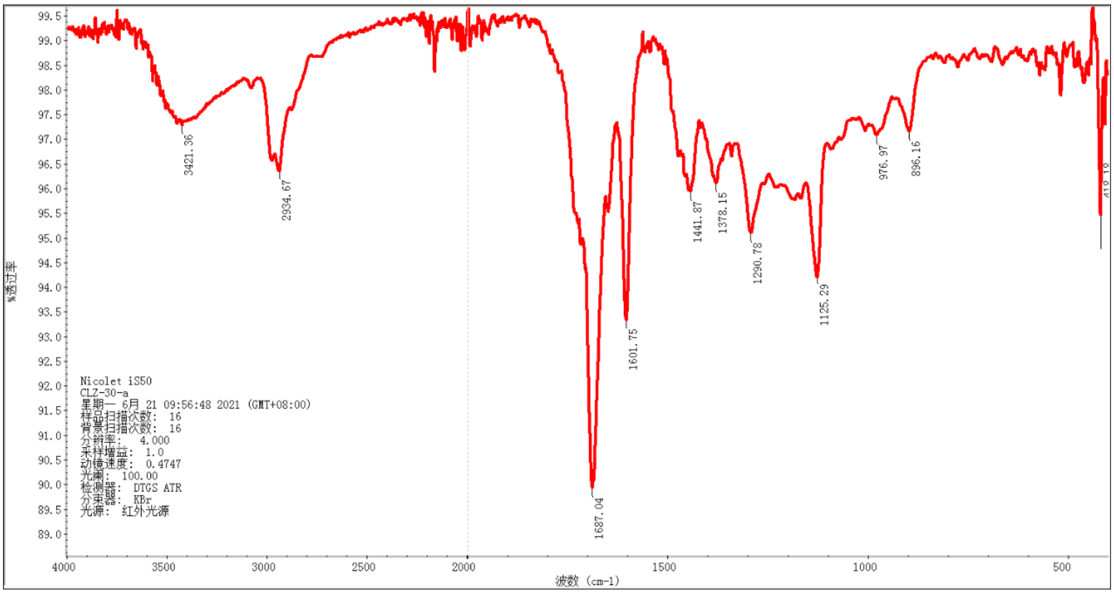

Figure S43. IR spectrum of **5**.

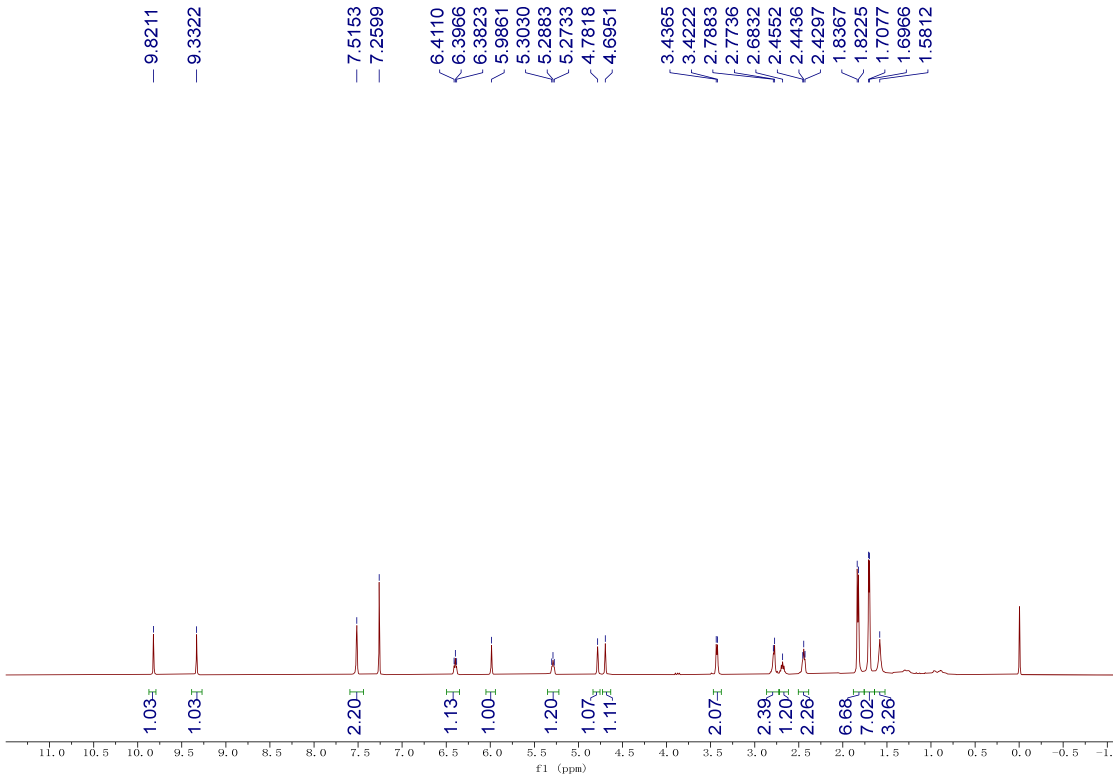

Figure S44. ^{1}H NMR (500 MHz) spectrum of **5** in $CDCl_3$.

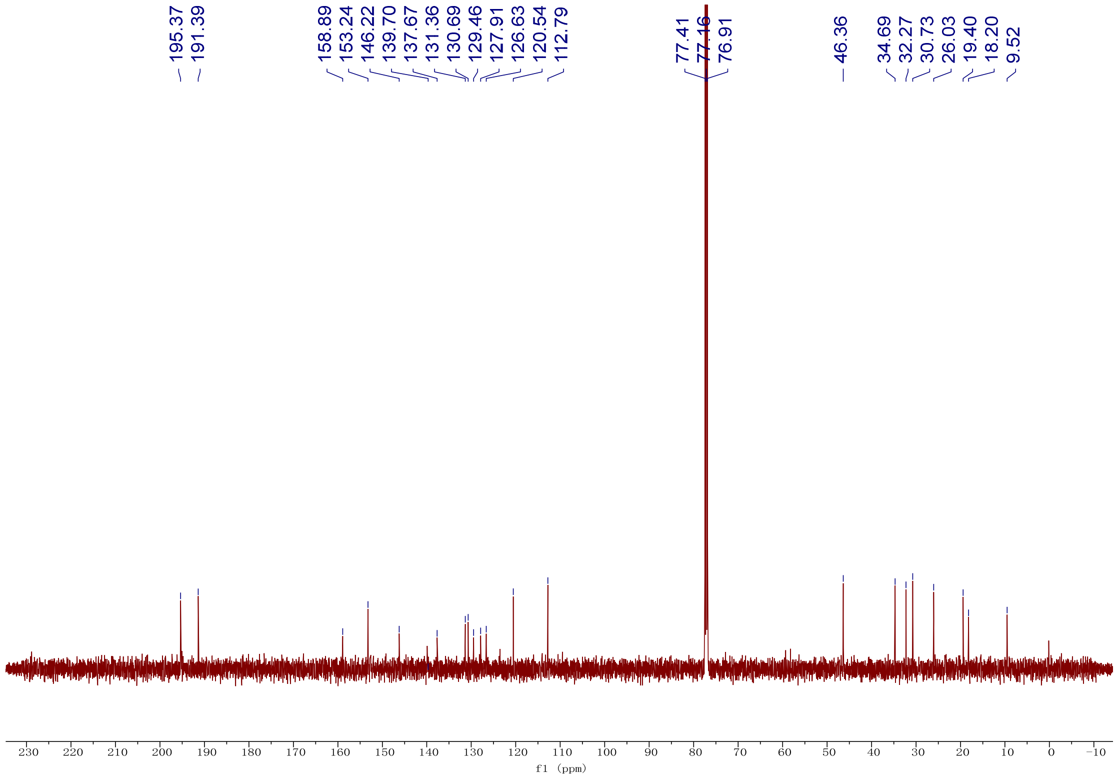

Figure S45. ^{13}C NMR (125 MHz) spectrum of **5** in $CDCl_3$.

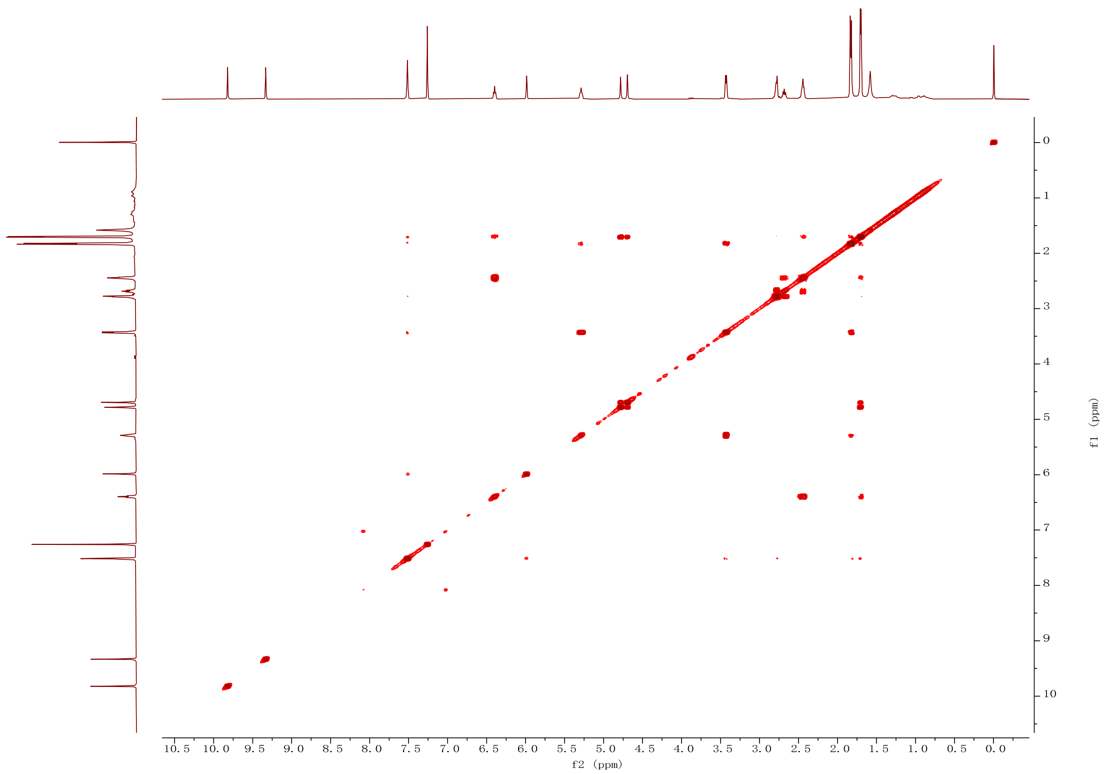

Figure S46. ^{1}H-^{1}H COSY (500 MHz) spectrum of **5** in $CDCl_3$.

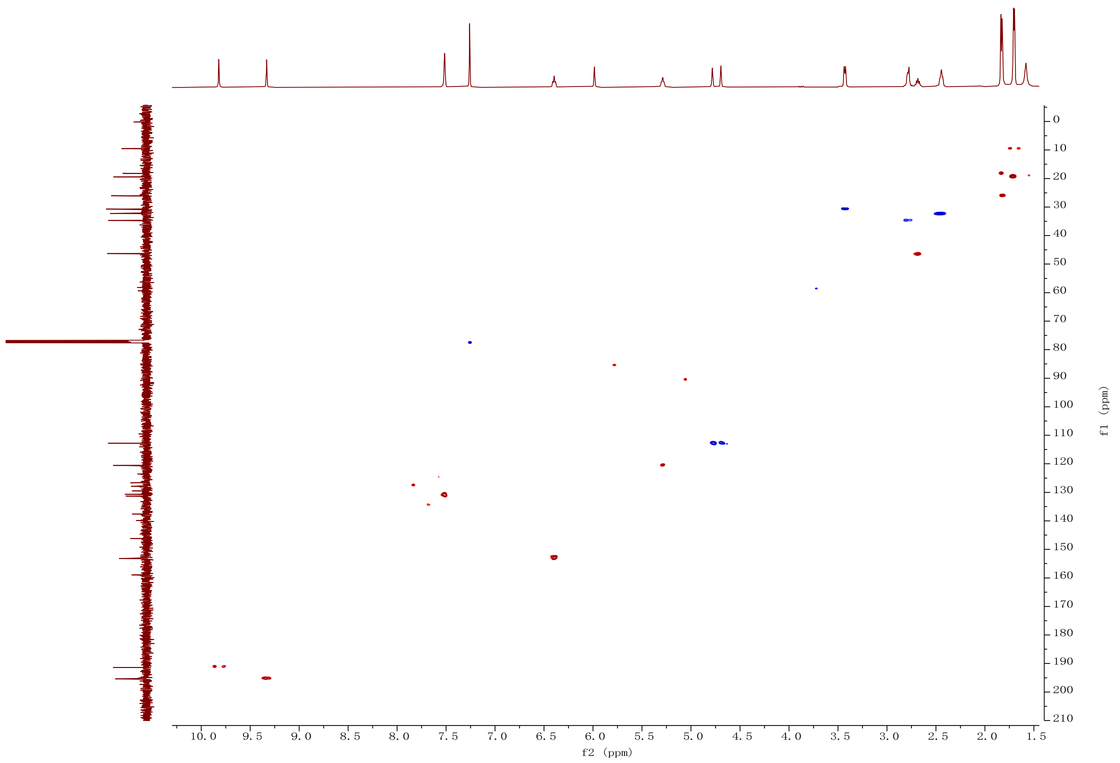

Figure S47. HSQC (500 MHz) spectrum of **5** in $CDCl_3$.

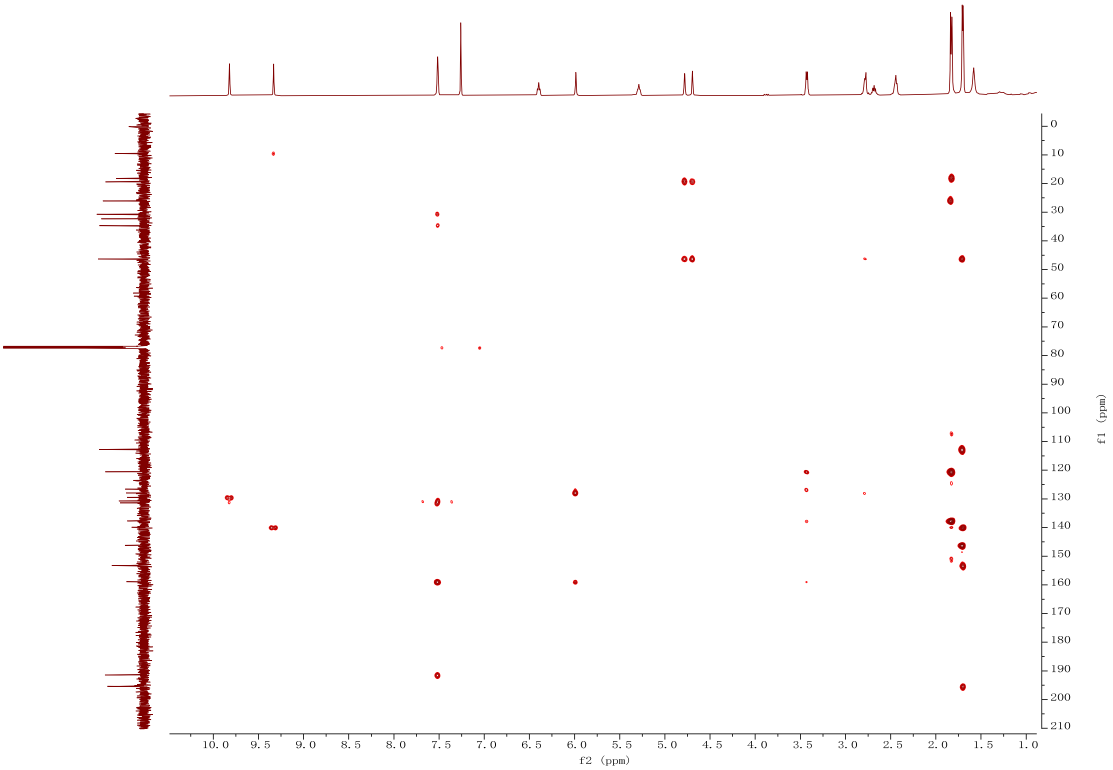

Figure S48. HMBC (500 MHz) spectrum of **5** in $CDCl_3$.

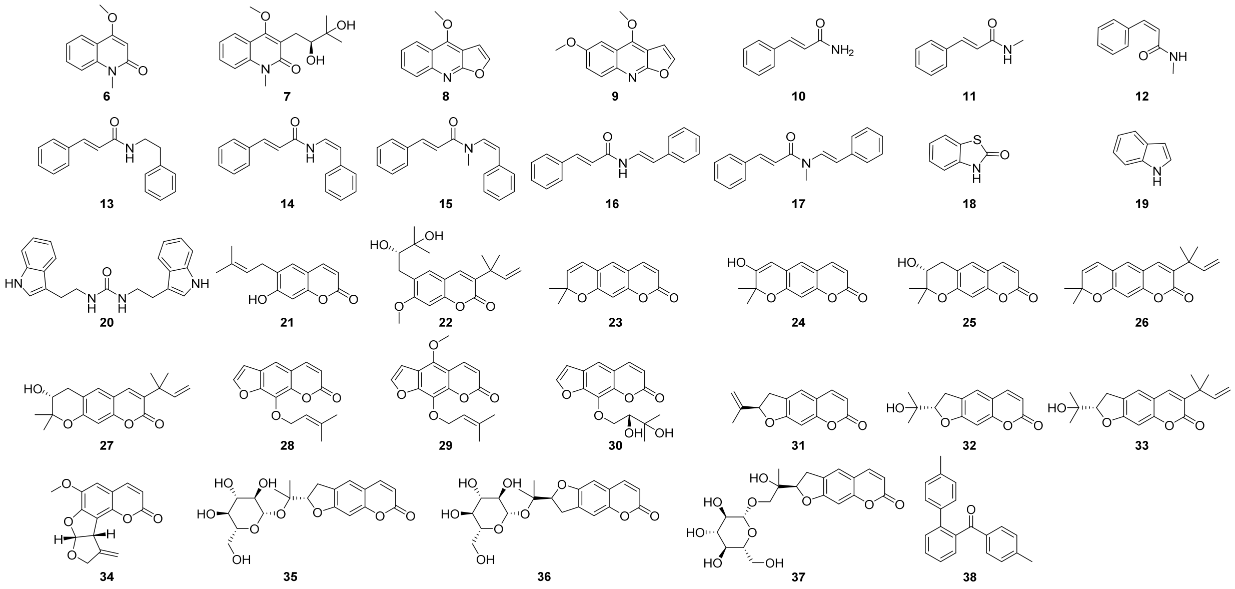

Figure S49. Chemical structures of compounds **6**−**38**.

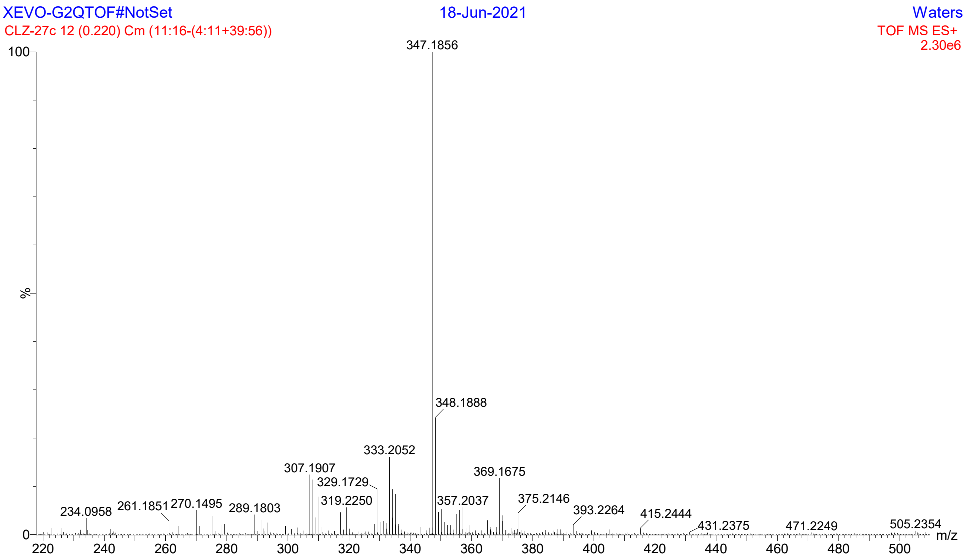

Figure S50. ESI-MS of **22**.

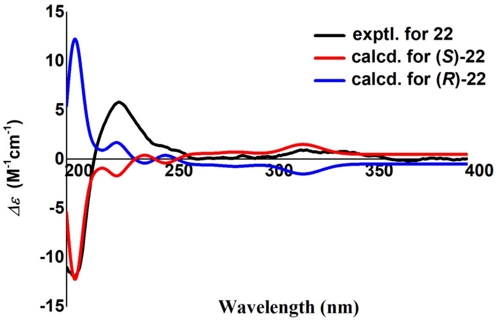

Figure S51. Experimental and calculated ECD spectra of **22**.
